# A Computational Model of Neoadjuvant PD-1 Inhibition in Non-Small Cell Lung Cancer

**DOI:** 10.1208/s12248-019-0350-x

**Published:** 2019-06-24

**Authors:** Mohammad Jafarnejad, Chang Gong, Edward Gabrielson, Imke H. Bartelink, Paolo Vicini, Bing Wang, Rajesh Narwal, Lorin Roskos, Aleksander S. Popel

**Affiliations:** 1 Department of Biomedical Engineering, Johns Hopkins University School of Medicine, Baltimore, Maryland USA; 2 The Sidney Kimmel Comprehensive Cancer Center, Johns Hopkins University School of Medicine, Baltimore, Maryland USA; 3 Department of Pathology, Johns Hopkins University School of Medicine, Baltimore, Maryland USA; 4 Clinical Pharmacology, Pharmacometrics and DMPK (CPD), MedImmune, South San Francisco, CA USA; 5 Department of Clinical Pharmacology and Pharmacy, Amsterdam UMC, Vrije Universiteit Amsterdam, Amsterdam, The Netherlands; 6 Clinical Pharmacology, Pharmacometrics and DMPK, MedImmune, Cambridge, UK; 7 Amador Bioscience Inc., Pleasanton, CA USA; 8 MedImmune, Gaithersburg, MD USA

**Keywords:** immune checkpoint inhibitors, immuno-oncology, immunotherapy, non-small cell lung cancer, quantitative systems pharmacology

## Abstract

Immunotherapy and immune checkpoint blocking antibodies such as anti-PD-1 are approved and significantly improve the survival of advanced non-small cell lung cancer (NSCLC) patients, but there has been little success in identifying biomarkers capable of separating the responders from non-responders before the onset of the therapy. In this study, we developed a quantitative system pharmacology (QSP) model to represent the anti-tumor immune response in human NSCLC that integrated our knowledge of tumor growth, antigen processing and presentation, T cell activation and distribution, antibody pharmacokinetics, and immune checkpoint dynamics. The model was calibrated with the available data and was used to identify potential biomarkers as well as patient-specific response based on the patient parameters. The model predicted that in addition to tumor mutational burden (TMB), a known biomarker for anti-PD-1 therapy in NSCLC, the number of effector T cells and regulatory T cells in the tumor and blood is a predictor of the responders. Furthermore, the model simulated a set of 12 patients with known TMB and MHC/antigen-binding affinity from a recent clinical trial (ClinicalTrials.gov number, NCT02259621) on neoadjuvant nivolumab therapy in resectable lung cancer and predicted an augmented durable response in patients with adjuvant nivolumab treatment in addition to the clinical trial protocol of neoadjuvant nivolumab treatment followed by resection. Overall, the model provides a valuable framework to model tumor immunity and response to immune checkpoint blockers to enhance biomarker discovery and performing virtual clinical trials to aid in design and interpretation of the current trials with fewer patients.

**Electronic supplementary material:**

The online version of this article (10.1208/s12248-019-0350-x) contains supplementary material, which is available to authorized users.

## Introduction

Lung cancer, predominantly non-small cell lung cancer (NSCLC), has been the leading cause of cancer-related mortality worldwide with consistently poor prognosis due to late diagnosis and lack of effective treatment strategies for late-stage cases. Chemotherapy and targeted therapies for NSCLC have shown to improve the survival, but often lack durable response. The approval of immune checkpoint blocking antibodies has revolutionized the treatment strategies for patients with advanced forms of lung cancer in the past few years (1). In particular, approved antibodies against PD-1 (nivolumab (2–4) and pembrolizumab (5–7)), PD-L1 (atezolizumab (8) and durvalumab (9)), and combination of nivolumab and anti-CTLA-4 (ipilimumab) (10) have significantly improved the overall survival of the advanced NSCLC patients. However, effective therapies that can replace or complement the current standard-of-care for early-stage NSCLC are lacking (11). A recent small clinical trial investigated the role of neoadjuvant nivolumab therapy for early-stage resectable NSCLC patients (11). Nivolumab treatment showed major pathological response in 45% of the resected tumor without delaying the surgery and resulted in expansion of T cell clones against the tumor antigens.

Despite the recent progress in immune checkpoint blockers, the predictive biomarkers able to efficiently stratify responders from non-responders are limited. Presence of PD-L1 is used as a biomarker for pembrolizumab in NSCLC (7), but it lacks specificity (12). Perhaps the most successful predictor of the responders thus far is identified as tumor mutational burden (TMB) based on whole-exome sequencing (13,14). In the neoadjuvant study described above, TMB was predictive of the responders (11). However, there are patients with high TMB that do not respond, and there are responders with low TMB. Thus, discovery of multimodal biomarkers is necessary to more accurately identify the potential responders, and computational models can complement and aid the clinical trials to achieve this goal.

Previous computational models have demonstrated the utility of model prediction in a variety of cases such as anti-angiogenic treatment for breast cancer (15), heterogeneity in anti-PD-1 therapy (16), dendritic cell therapy in melanoma (17), immunogenicity of therapeutics (18,19), and combination of radiation and anti-PD-1 therapy in mouse colon cancer (20). Specifically, quantitative systems pharmacology (QSP) models capable of integrating our knowledge of cancer biology and immunology across multiple spatial and temporal scales have proven to be necessary in describing the complex cycle of anti-tumor immune response (16,20–22). Although recent studies have provided valuable insight in specific cases, primarily studied in mice, a human-centric mechanistic model based on the clinically measured patient characteristics (e.g., TMB, mutational landscape, MHC/antigen binding strength) is lacking.

Here, we constructed a quantitative systems pharmacology model to describe the anti-tumor immune response for NSCLC in human and investigated the role of adjuvant and neoadjuvant anti-PD-1 treatment for early-stage NSCLC. The model includes important features such as tumor growth, detailed representation of the antigen processing and presentation by mature antigen presenting cells (mAPC), migration of the mAPC to tumor-draining lymph node(s) (TdLN), T cell priming, egress and distribution of effector T cells (Teff) to the tumor and the rest of the body, PD-1/PD-L1 axis between Teff and cancer cells, as well as inhibitory mechanisms through regulatory T cells (Treg). Overall, the model aims to provide understanding of the complex processes that drive effective anti-tumor immune response to provide novel directions for clinical research and biomarker discovery.

## Methods

### Computational Model Structure

**Fig. 1:**
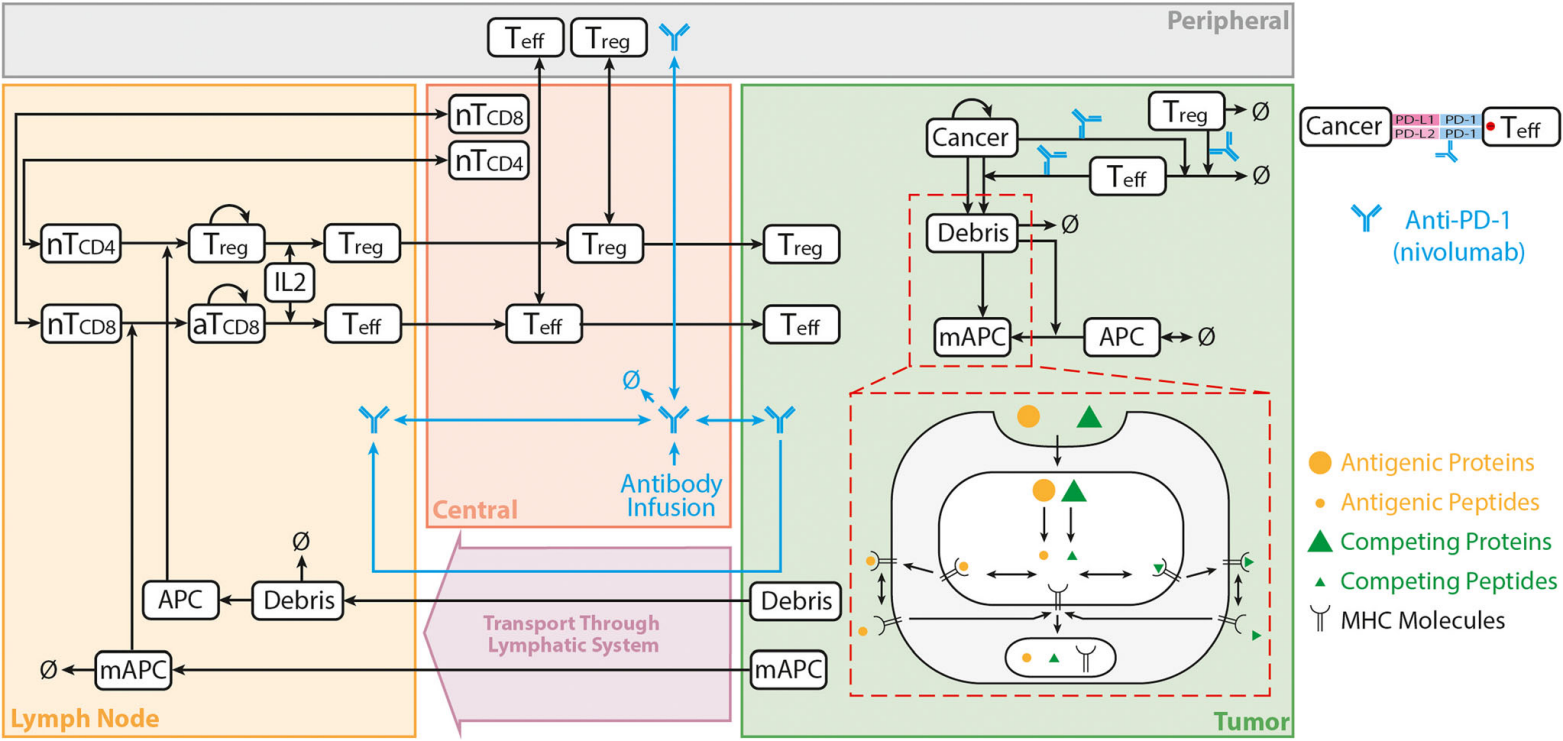
Diagram of the main cellular and molecular interactions implemented in the model. The diagram illustrates compartments, cellular, and inter- and intracellular interactions as well as antibody pharmacokinetic. Cancer cell death in the tumor leads to release of antigen and activation of APC that mature, pick up the antigen, and migrate to the TdLN to activate Teff response. Additionally, the antigen drains to the TdLN and is presented by immature APC to induce Treg. Teff and Treg are distributed to the tumor to enhance cancer cell killing by Teff or reduce it through inhibition by Treg response. Anti-PD-1 antibody nivolumab in the tumor affects the rate of cancer cell killing by Teff and also exhaustion of Teff by cancer cells

The model was developed to capture the essential features of anti-tumor immune response important in anti-PD-1 therapy in the context of NSCLC (Fig. 1). While the governing equations are explained in detail in the supplementary information, we briefly describe the interactions that have been included in the model. In this model, cancer cells grow and die in the basal condition, which results in the release of self and cancer-associated antigens. The antigen is picked up by mAPC (primarily dendritic cells), processed into peptides, and presented on the cell surface through major histocompatibility complex (MHC) molecules. This part of the model has detailed molecular components to be able to utilize the quantified binding affinity of antigenic peptides. These mAPC then upregulate C-C chemokine receptor 7 (CCR7) to migrate through lymphatic vessels to the TdLN where they can activate naïve CD8 T cells to transform them to activate and eventually effector T cells, Teff. Additionally, the immature antigen presenting cells (APCs) in the LN pick up the soluble antigen and induce regulatory T cells, Treg. Teff and Treg then exit the LN and traffic to the blood and, after extravasation, are distributed to tumor as well as other peripheral tissues. Teff in the tumor contribute to cancer cell killing and can be exhausted by Treg and PD-1/PD-L1-mediated interactions with cancer cells. Antibody transport was modeled using a validated physiology-based model previously described in detail (15) and is used to model transport of nivolumab in this study. It should be noted that although nivolumab parameters are used in this study, the model is applicable to any checkpoint inhibitor. The four-compartment model comprising tumor, TdLN, central (blood), and peripheral (all other organs and tissues) compartments, thus representing the entire patient, is formulated in the form of ordinary differential equations (ODE) and algebraic equations; the current version comprises 55 ODE and 53 algebraic equations. SimBiology platform in MATLAB R2018b (MathWorks) was used for all the simulations and sensitivity analysis. Lists of compartments, species, parameters, reactions, rules, and events of the model as well as the Systems Biology Markup Language (SBML) version of the computer code are presented as Supplementary Material.

### Parameter Sensitivity Analysis

For complex computational models, it has become a standard practice to conduct parameter sensitivity analysis (PSA) to determine which parameters of the model have a high impact on the variables of interest (e.g., tumor volume or diameter in our study) and rank the parameters in order of the impact and which parameters have a low impact. Latin hypercube sampling (LHS) along with a log-normal transformation was used to vary 30 parameters simultaneously to investigate the effect of model inputs on the model outcome namely tumor diameter, percent change in tumor diameter, number and density of Teff and Treg, ratio of Teff to Treg in tumor, and T cell clonality in the blood. A sample of size 2000 was chosen and the effect of sample size was assessed by calculated top-down coefficient of concordance for the predictions; the coefficient for two subsequent sets is 0.933 (23,24). The selected input parameters and the range of their variation are listed in Table S1. Partial rank correlation coefficient (PRCC) was used to identify the most influential model inputs on the results (23). Significance of the correlations is reported in the Supplementary Figure S1 in the form of heatmap.

### Clinical Trial Data Used in the Model

The model was developed with the data from neoadjuvant nivolumab (anti-PD-1) clinical trial in NSCLC in mind (11) (ClinicalTrials.gov number, NCT02259621). Briefly, patients with untreated early-stage (I, II, and IIIa) surgically resectable NSCLC tumors were treated with two doses of 3 mg/kg nivolumab before surgery. Tumor size was measured before treatment and before surgery (approximately 4 weeks after initial dose) using computed tomography. Additionally, whole-exome sequencing was performed on pre-treatment biopsies to quantify tumor mutational burden, identify tumor antigens, and their MHC binding affinity as well PD-L1 status of the tumor. Tumor mutational burden (as a measure of anti-tumor T cell clones) and binding affinity of the antigen were directly used in simulating patient-specific response. The other 28 parameters that were varied in PSA were randomly sampled from a log-normal distribution with half the geometric standard deviations of what was used for PSA. The log-normal distribution of the parameters was assumed for all the parameters due to the limited information on the distributions. Sample size of 200 was used for individual patients, and the effect of sample size was assessed by two-sided Wilcoxon rank sum test of two subsequent sampling of size 200 and showed no significant difference for any of the patients.

### Statistical Analysis

Comparison between multiple groups was done using a non-parametric method, Kruskal-Wallis test, followed by Bonferroni correction to adjust for multiple comparison. Wilcoxon signed-rank test was used to compare the distribution of the regression predicted by the model with the pathological quantification of the resected tumors from the trial (25). MATLAB R2017b (MathWorks) was used for the statistical analysis.

## Results

### Presentation of Antigen by Antigen Presenting Cells

**Fig. 2:**
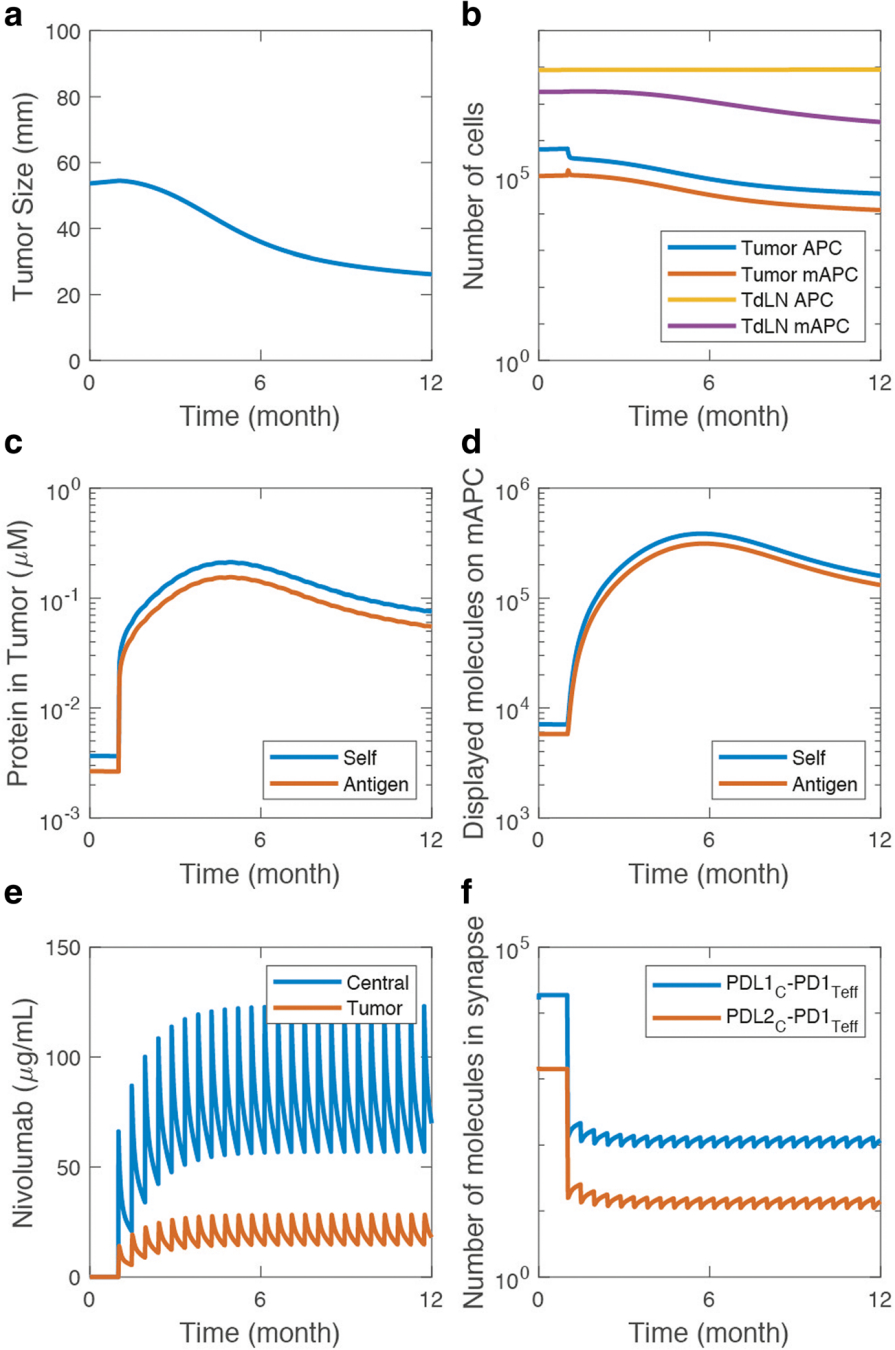
Details of antigen presentation and nivolumab treatment. The sample illustrates a case with response to nivolumab treatment evident by the decrease in the tumor size (**a**). Death of cancer cells activates APCs in the tumor where they pick up antigen and migrate to the TdLN (**b**). The amount of antigenic and self proteins in the tumor microenvironment (**c**) along with the affinity of peptide to MHC binding defines how many of each type of peptides are presented on the mAPC (**d**). The amount of PD-1/PD-L1 and PD-1/PD-L2 interactions in the immunological synapse between Teff and cancer cells (**f**) is used to determine Teff killing and Teff exhaustion by Treg and cancer cells, which is also modulated by nivolumab concentration in the tumor (**e**). All panels show the results from a single simulation with biweekly nivolumab treatment starting at 1 month

The model is able to connect the chain of events from tumor growth to antigen release by immunogenic death of cancer cells, APC maturation, and antigen processing and presentation by mAPC, to build understanding of how these processes regulate anti-tumor immune response and affect treatment strategies (Fig. 2). An example case of growing tumor treated with biweekly nivolumab dosing in Fig. 2 shows how the tumor size is reduced as a result of anti-tumor T cell response (Fig. 2a). The number of mAPCs increases in the tumor as a result of immunogenic cancer cell death, and secondly in the TdLN wherein presumably, T cells are activated (Fig. 2b). The dying cancer cells release self and antigenic proteins in the tumor microenvironment where mAPCs can engulf the proteins (Fig. 2c), break them down into peptides and present them (Fig. 2d). The relative concentration of these released proteins as well as the MHC binding affinity of individual peptides eventually determines how many of each type of peptides are presented on the surface of the mAPC (Fig. 2d), which eventually determines how efficiently the mAPCs that migrate to TdLN would be able to initiate T cell response. For the purpose of this study, we primarily investigated the effect of anti-PD-1 therapy (nivolumab) on the immune response against NSCLC. In the model, nivolumab was administered through central infusion and was transported to tumor as well as healthy tissues (Fig. 2e). In the tumor microenvironment, nivolumab binds and blocks PD-1 on the surface of the T cells, which results in the fewer PD-1/PD-L1 and PD-1/PD-L2 engagements in the immunological synapse between T cells and cancer cells (Fig. 2f). This leads to higher levels of T cells killing and lower levels of T cell exhaustion in the tumor microenvironment due to diminished negative signal. Although a specific partial responder case was presented in this section, in the next section, we explore a variety of responses that the model is able to exhibit.

### Variety of Anti-Tumor Immune Responses Captured by the Model

**Fig. 3:**
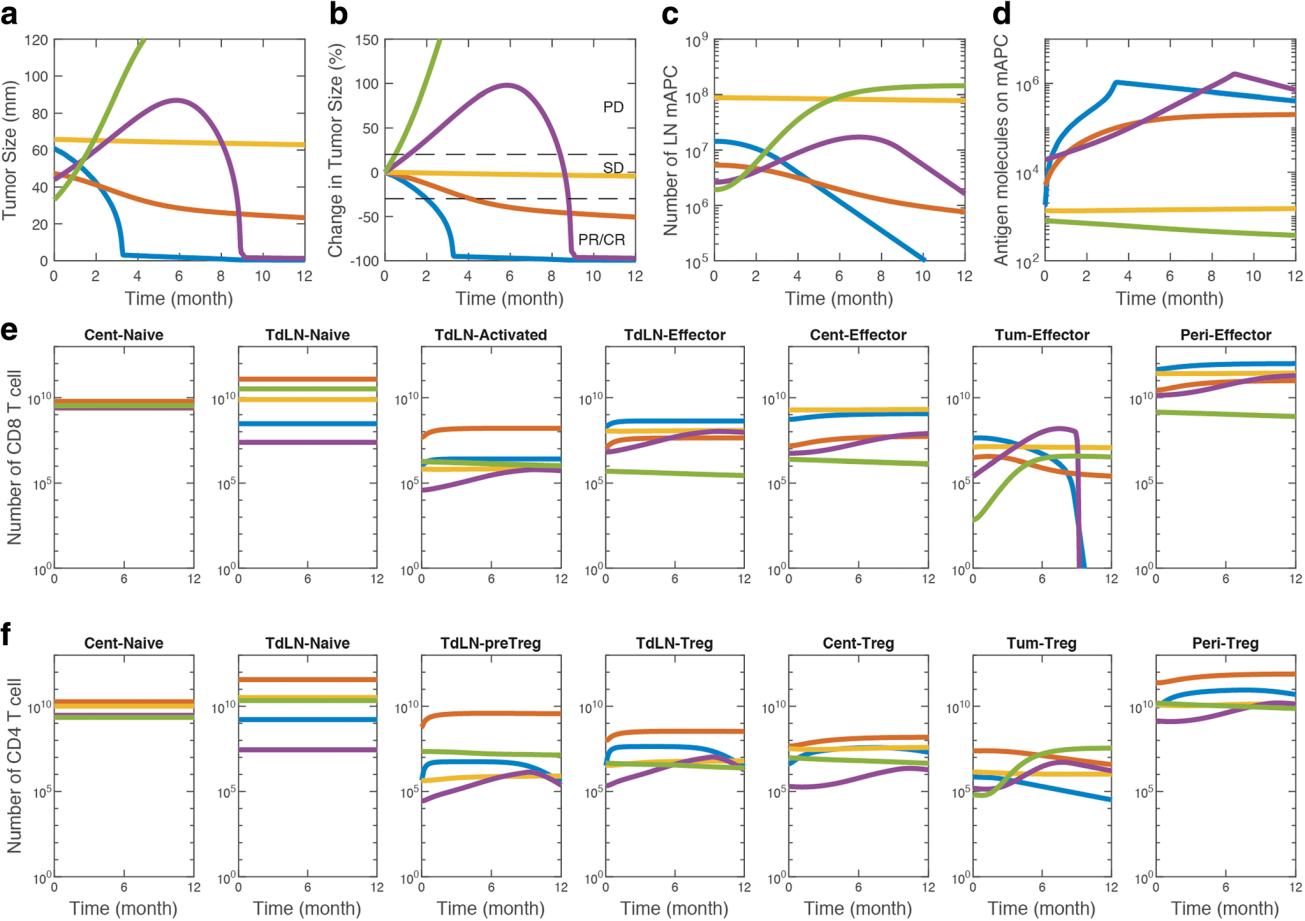
Diversity of response captured by the QSP model. The model is able to capture a variety of responses similar to the ones observed in clinical trials for different sets of model parameters or initial conditions. Changes in the tumor size (**a**) and percent change in tumor size (**b**) shows responses in 1 year period that correspond to partial or complete response (PR/CR), stable disease (SD), or progressive disease (PD) based on RECIST criteria. Important elements of the model such as number of mAPC in TdLN (**c**), number of antigens presented on mAPC (**d**), and dynamics of Teff (**e**) and Treg (**f**) populations in all compartments can be extracted and used to predict the responder characteristics. Traces of same color represent different components of the same simulation

The model is capable of capturing a variety of responses that depend on initial conditions and model parameters as illustrated in Fig. 3. The model is able to exhibit RECIST category responses such as complete response (CR, blue), partial response (PR, red), stable disease (SD, yellow), progressive disease (PD, green), and specific cases such as pseudo-progression (purple) shown in Fig. 3a, b. The fact that these cases were produced without changing the structure of the model and only by varying some of the model parameters and initial conditions suggests that current structure is capable of capturing the breadth of the clinical responses, which depends on the individual patient and tumor characteristics. In addition to the tumor diameter, each case results in specific dynamics of mAPCs in the TdLN (Fig. 3c) as well as number of antigenic peptides displayed on mAPC (Fig. 3d). As explained in previous section, these two important factors along with the availability of T cells and associated factors define the extent of anti-tumor T cell response that is separated into Teff (Fig. 3e) and Treg (Fig. 3f) responses. In these samples, the responders emerge with higher numbers of Teff compared to Treg in the tumor which leads to effective cancer cell killing and tumor shrinkage. The PD case depicted here in green (Fig. 3d) demonstrates how a weak antigen that leads to few antigenic peptides displayed on mAPC results in a limited Teff response that is not sufficient for tumor eradication. To identify relative importance of the parameters in separating the responders from non-responders, we performed parameter sensitivity analysis presented in the next section.

### Identification of the Important Parameters in Anti-Tumor Immune Response

**Fig. 4:**
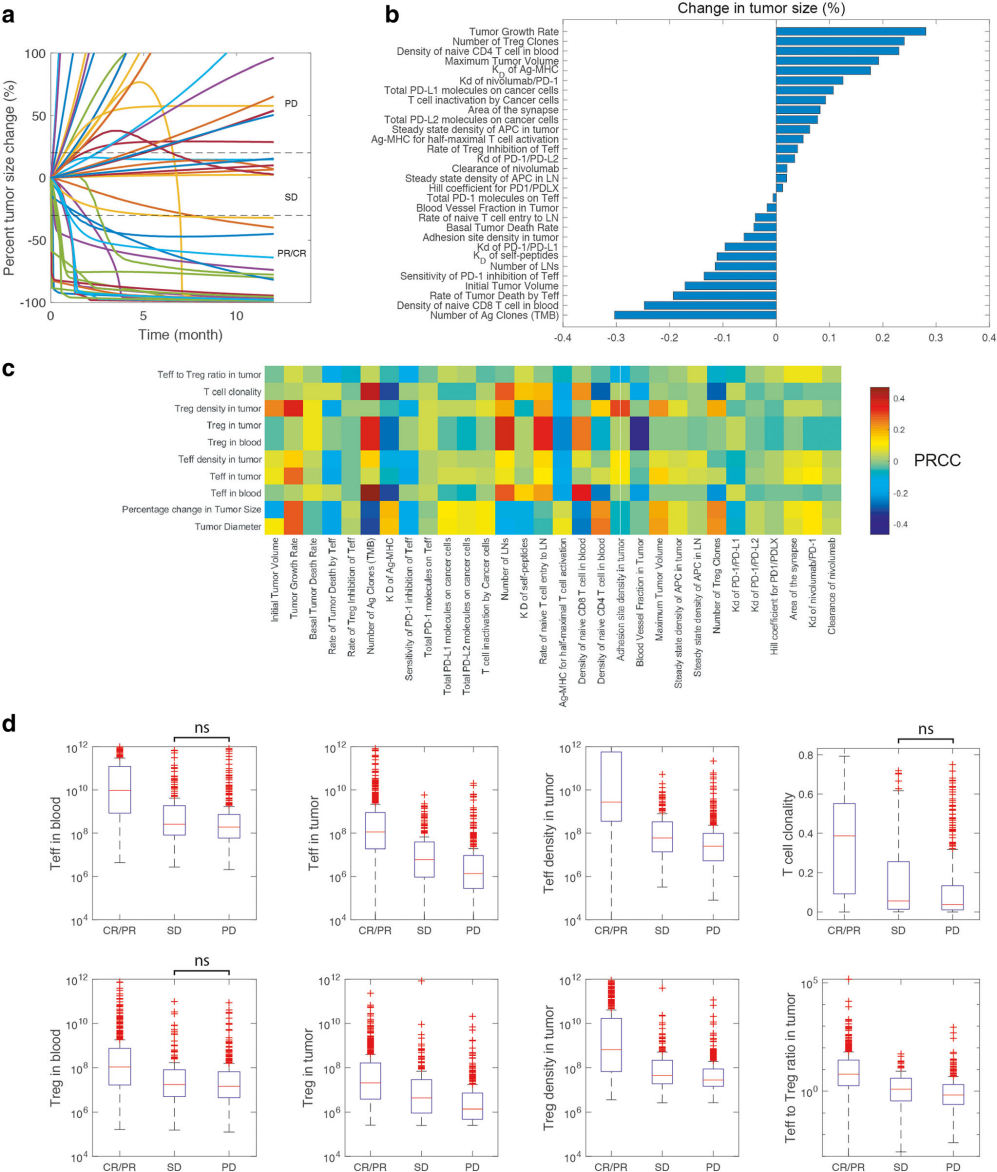
Parameter sensitivity analysis. Sensitivity analysis was performed by varying a set of 30 parameters simultaneously and performing partial correlation analysis to find out the effect of those inputs on the model outputs, primarily percent change in the tumor size (**b**). Data shown in **b** is from analysis of 2000 simulations set using Latin hypercube sampling, although only 100 random traces are shown in **a**. The partial rank correlation coefficient, PRCC, for individual parameters with outputs is provided in **c**. The response in the 2000 simulations was stratified into CR/PR, SD, and PD, and statistical comparisons were made between the groups for output parameters (**d**). All the comparisons showed differences that were statistically significant (*p* < 0.01 for corrected Kruskal-Wallis test) unless stated as not-significant (ns)

The contribution of primary model parameters to the changes in the tumor size is investigated using parameter sensitivity analysis for cases with biweekly nivolumab treatment for 1 year (Fig. 4). Table S1 lists a set of 30 parameters of the model alongside the geometric standard deviation in which they were varied for sensitivity analysis. Simultaneous changes of these parameters resulted in a range of responses that covered all possible clinical outcomes, i.e., PR/CR, SD, and PD (Fig. 4a). Among the model parameters, TMB, density of naïve CD8 T cells in blood, and rate of cancer cell death by Teff were the top parameters that correlated with the smaller tumor diameters (Fig. 4b). Conversely, tumor growth rate, number of Treg clones, and density of naïve CD4 T cells in the blood correlated with percentage increase in tumor size (Fig. 4b). Tracking the individual cases revealed that about 23% of the cases were cancer-free after 1 year treatment with nivolumab (Fig. 4c). It is important to distinguish between the cases presented here and individual patients that present themselves in the clinic. The goal of this parameter sensitivity analysis is to find the important parameters of the model affecting the output, whether the parameters can represent direct patient-specific measurables (e.g., tumor mutational burden) or not (e.g., rate of cancer cell death by Teff). In addition to percent change in tumor diameter, the effect of varying these 30 parameters on other measurable model outputs such as CD8 T cell clonality in blood as well as number and density of Teff, Treg, and their ratio was investigated by calculating the PRCC (Fig. 4d; Figure S1). Parameters that were important in Teff and Treg response were TMB, rate of naïve T cell entry to TdLN, number of TdLN, and blood vessel density in the tumor. Similar parameters appeared to affect the CD8 T cell clonality in the blood. Additionally, the cases were stratified based on their percent change in tumor size and RECIST criteria into CR/PR, SD, and PD categories (Fig. 4e). The cases in CR/PR had significantly higher Teff and Treg in the blood and the tumor. Furthermore, T cell clonality and Teff to Treg ratio were higher in CR/PR compared to SD and PD.

### Relative Contribution of TMB and MHC/Antigen Affinity in Response

**Fig. 5:**
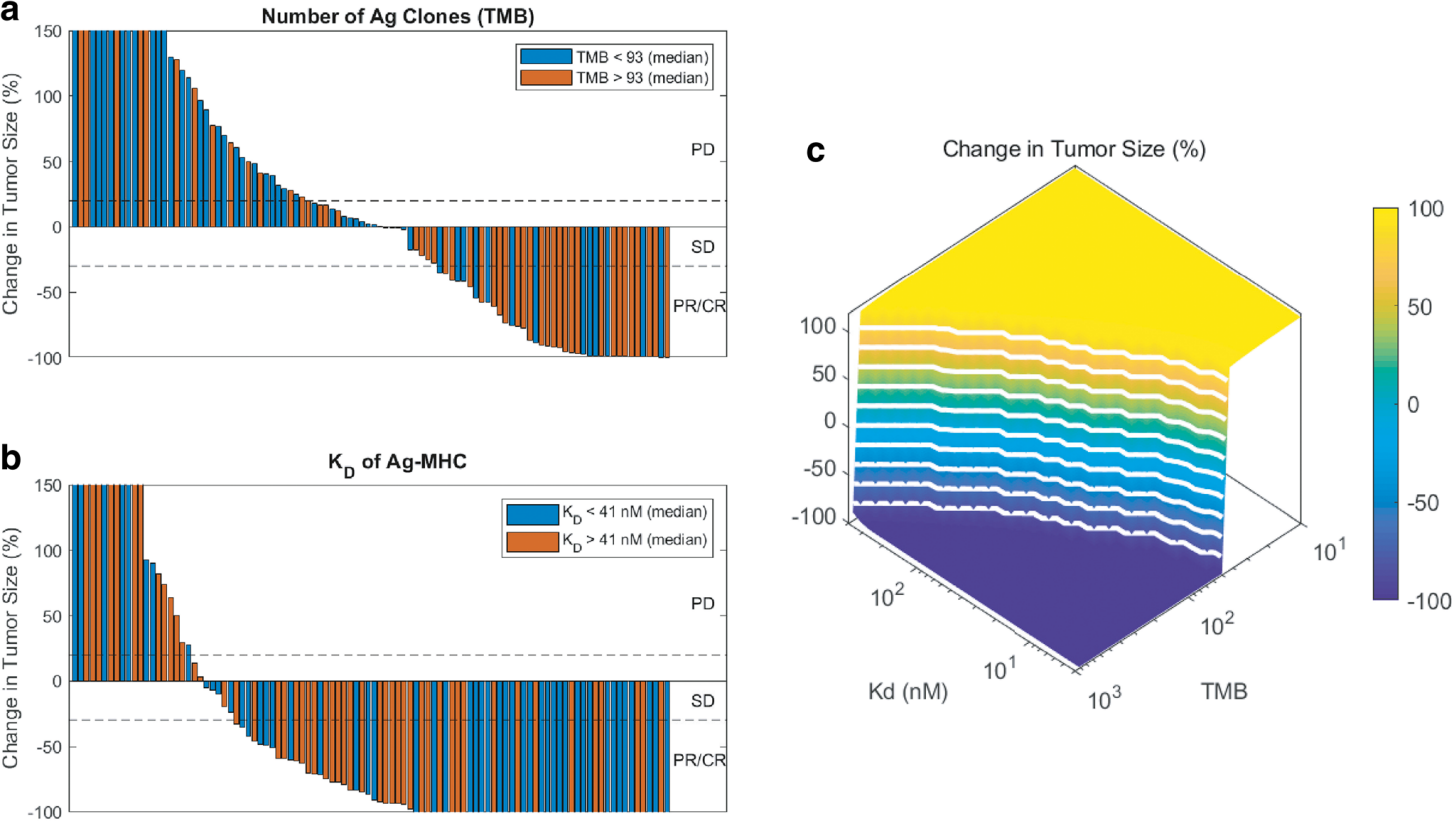
Variation of TMB vs MHC/antigen affinity. Waterfall plots for 100 simulations while varying TMB (**a**) or MHC/antigen affinity (**b**) with random variation of the rest of 30 parameters depicts the change in tumor size at 1 year period in response to nivolumab treatment. Percent change in tumor size for simultaneous variation of TMB and MHC/antigen affinity showed regions of response close to high TMB and low K_d_ (**c**)

Two patient-specific parameters have been measured in recent clinical trials to be examined as biomarkers for anti-PD-1 treatment; first, number of mutations in the tumor (or TMB) (14), and second, sequences of tumor-associated mutations that can be translated to MHC/antigen binding based on the sequence similarity to known antigen binding using artificial neural network software packages such as netMHCpan (26). To investigate the effect of these two important parameters, we varied them individually. Higher TMB correlated with earlier response (Fig. 5a), while higher K_d_ of MHC/antigen binding led to worse outcome or larger tumor diameters (Fig. 5b). Waterfall plots better show that in the clinically relevant ranges, higher TMB correlates with lower tumor diameters (aggregation of red bars on the right-hand side in Fig. 5a). Variation of antigen affinity in the clinical range revealed that complete responders often exhibit strong antigens (Fig. 5b). Varying these two parameters simultaneously under the baseline case demonstrated that lower diameters are achieved under combinations of lower MHC/antigen K_d_ and higher TMB (Fig. 5c).

### Model Prediction of Patient-Specific Outcome Under Adjuvant and Neoadjuvant Anti-PD-1 Therapy

**Fig. 6:**
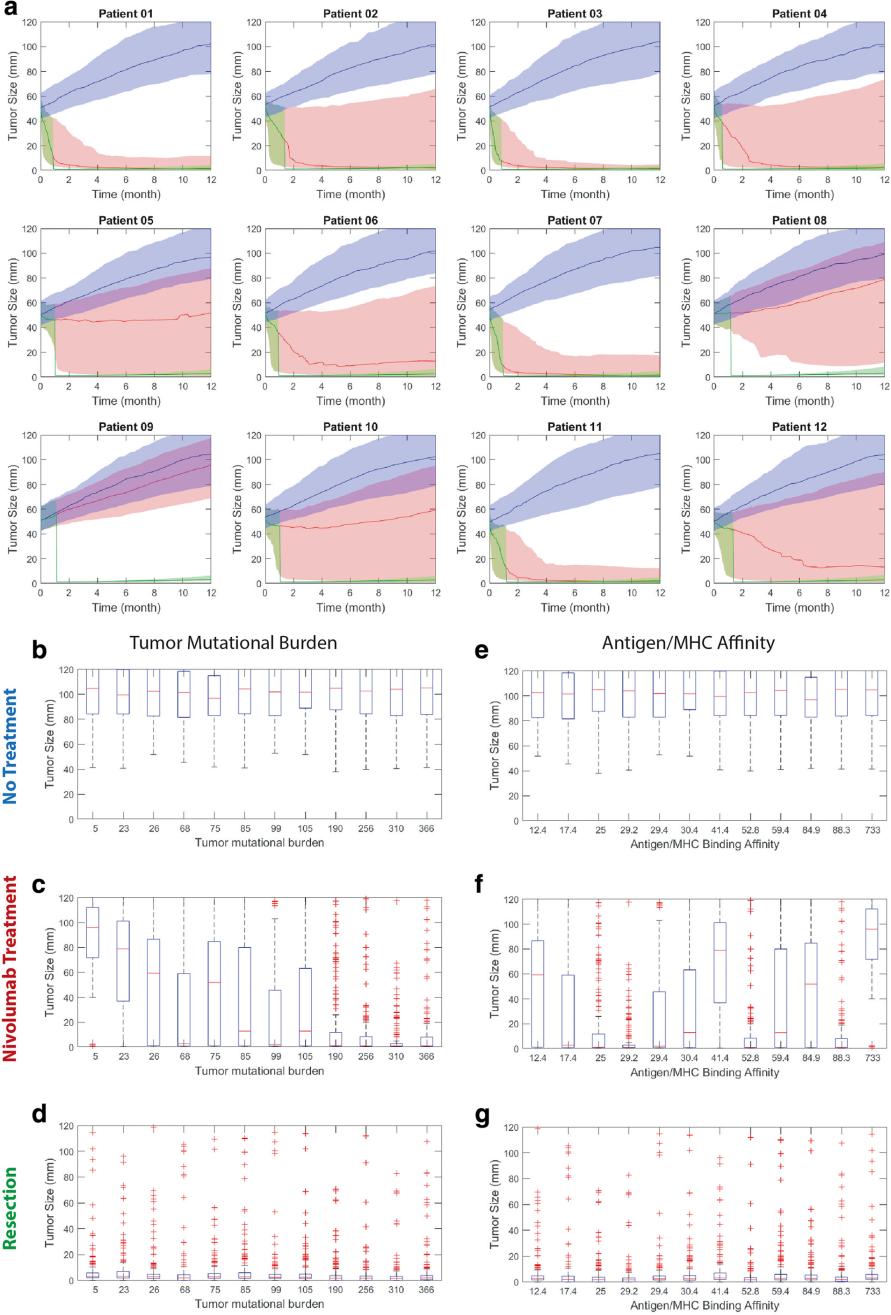
Patient response to nivolumab. TMB and MHC/antigen affinity were set according to the measured patient data, and the rest of the 25 parameters (same parameters in PSA) were randomly changed based on Latin hypercube sampling for 200 simulations/patient (**a**). Three scenarios were explored under no treatment (blue), biweekly nivolumab treatment (red), and two doses of nivolumab followed by resection. The data is reported as median ± 60% prediction interval. Model suggests higher TMB correlates with better response, whereas MHC/antigen affinity in clinical range is not sufficient for improved response. Under all three conditions of no treatment (**b**, **e**), nivolumab (**c**, **f**), and nivolumab + resection (**d**, **g**), higher TMB correlated with better response in terms of tumor diameter. TMB or MHC/antigen affinity-sorted patient data from **a** at 1 year are shown using boxplot

TMB and MHC/antigen affinity of 12 patients were measured (Table S2) in a recent small clinical trial to examine effect of nivolumab in resectable lung cancer (ClinicalTrials.gov number, NCT02259621; the data reported in (11) were used in our simulations). Model simulations were performed by setting these two measured parameters for the patients while varying the rest of the parameters in Table S1 for 200 cases to account for our uncertainty in knowing the rest of the parameters related to the patient (Fig. 6). Taking a different viewpoint, we consider a cohort of virtual patients whose two measured characteristics are identical to those of the patients in the clinical trial, but other (not measured) characteristics vary; we then conduct virtual in silico trials with these patients. Three explored scenarios were no treatment (blue in Fig. 6), biweekly nivolumab treatment for a year (red in Fig. 6), and two doses of nivolumab followed by resection where a presumed 1 mm^3^ nodule remained with similar proportions of cancer and immune cells (green in Fig. 6). Overall, resection appeared to be the most effective and consistent way of reducing the tumor diameter, whereas biweekly nivolumab was able to shift the probabilities towards smaller diameters in a patient-specific manner. Based on the model predictions and according to the 60% (20% to 80% range) prediction interval (PI), none of the patients had a chance of sufficient endogenous immune response to eradicate the tumor on their own and without any treatment (Figure S2). Patients 1, 2, 3, 4, 7, and 11 showed largest tumor size reduction with biweekly nivolumab treatment evident by the 1-year median diameters of close to zero (Figure S3). Resection showed consistent reduction in the tumor diameter, but the simulations suggest that a small residual nodule can grow back in longer time points after 1 year (Figure S4).

### Tumor Mutational Burden Is a Reliable Biomarker

Model findings from the in silico trial explored in previous section confirms the conclusions of the clinical trial that tumor mutational burden is a dominant biomarker to separate responders from non-responders, and also suggests that MHC/antigen affinity did not demonstrate any trends for the majority of the patients except in extreme clinical cases (Fig. 6b–g). Ordering the patients based on their TMB revealed a clear trend in 1-year median tumor diameter. For biweekly nivolumab treatment, patients with TMB of higher than 190 total sequence alterations showed consistent near zero median diameters, in contrast to TMB values lower than 26 which had diameters near maximum tumor diameters. The resection appeared to have similar effect between the patients. This is perhaps because the assumed 1 mm^3^ remaining nodule if not completely removed by the anti-tumor immune response will grow based on the tumor growth rate. MHC/antigen affinity on the other hand showed no apparent correlation between patients, primarily because the 11 out of 12 patients had affinities of the same order of magnitude (12.4 to 88.3 nM). The exception was patient 9 that had an MHC/antigen affinity of 733 nM, which showed the highest median tumor diameter. Similar to no-treatment group, there was no trend except for patient 9. The patients had very similar profiles in the resection treated group.

**Fig. 7:**
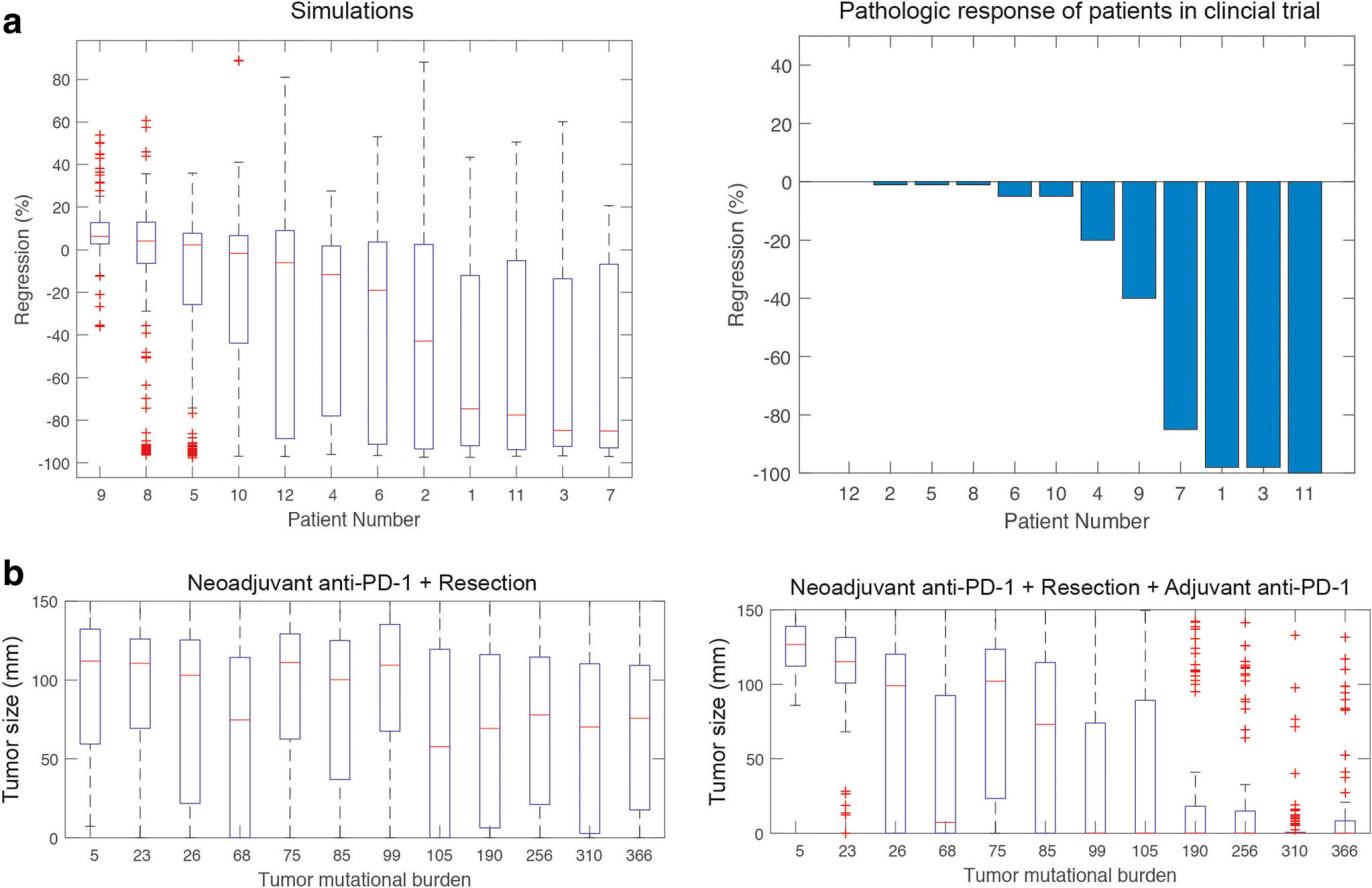
Model predicts additional benefit from adjuvant anti-PD-1 treatment for high TMB patients. **a** Comparing regression response for simulated patients at the time of resection (~ 40 days) for patients from Fig. 6a with regression based on pathologic response of patients in clinical trial showed that responders based on the model (patients 7, 3, 11, and 1) correlate with clinical data (Wilcoxon signed-rank test, *p* value = 0.765). The tumor size at 5 years after surgery was compared for “neoadjuvant anti-PD-1 + resection” and “neoadjuvant anti-PD-1 + resection + adjuvant anti-PD-1” (**b**). Model predicts that addition of adjuvant anti-PD-1 therapy improves the response in patients with high TMB. Simulated patients here have the same characteristics as the previous analyses in this work (Fig. 6). Boxplots show the results from 200 simulations per patient

A more direct comparison could be made between the percentage tumor regression quantified by pathologic assessment of the resected patient samples, and the results from the resection simulations of the in silico model (Fig. 7a). Model prediction of the regression during the period before the surgery correlated well with the reported pathological regression measured from the resected samples from the trial (Fig. 7a). Wilcoxon signed-rank test between the model prediction of regression and the clinical data showed no significant difference between the two distributions (*p* value = 0.765) demonstrating that this fit-for-purpose model predicts the observed regression in the tumors. Additionally, the model predicts that in cases with undetected metastatic lesions, neoadjuvant anti-PD-1 treatment followed by resection would not mount sufficient anti-tumor immune response to clear the metastatic lesions (Fig. 7b). We investigated long-term tumor burden (tumor size 5 years after surgery) in a hypothetical trial that included similar patients to Forde *et al.* trial (11) in which these patients received adjuvant anti-PD-1 dosing after neoadjuvant anti-PD-1 and resection. Simulated patients who received adjuvant anti-PD-1 who also had high TMB were able to clear the metastatic lesion (Fig. 7b—left panel). However, patients that only received neoadjuvant treatment with resection even with high TMB were not able to overcome the metastatic nodule (Fig. 7b—right panel).

### Model Predicts Continuous Dosing Necessary for Optimal Response

The variation of dosing scheme showed that small variations in the three parameters of number of doses, amount per dose, and dosing interval do not change the response to anti-PD-1 therapy (Figures S5 and S6). Three, 6, and 12-month dosing periods were tested and the model predicted that the continuous dosing slightly improves reduction in tumor size at 1-year. Higher doses of 10 mg/kg and shorter dosing interval appeared to slightly enhance the median and the range of the response (Figures S5 and S6); however, none of the explored dosing schemes resulted in statistically significant changes (Figure S6). Higher doses and shorter dosing interval are both known to increase the side effects from the anti-PD-1 therapy (27).

## Discussion

Despite the remarkable success of immune checkpoint inhibitors in clinical trials, our understanding of the intricacies associated with anti-tumor immune response is limited. The quantitative systems pharmacology modeling offers valuable insight by integrating various experimental and clinical data to enhance our understanding of the cancer growth and anti-tumor immune response. The model presented in this study aims at including many important biological processes such as cancer cell growth, antigen release, antigen processing and presentation by APC, T cell activation, proliferation and infiltration to tumor, cancer cell killing, and mechanisms of T cell inhibition and exhaustion. In particular, the model includes a detailed expression of the antigen presentation that allows us to directly use patient-specific antigen strength data available from recent clinical trials (11,28). The model was developed and parameterized based on a variety of experimental and clinical data in the literature with extensive emphasis on the use of the data from human sources to build confidence on the use of the model for clinical trials (11,29–31). The model showed to be capable of capturing the variety of the responses observed in the clinical trials. In particular, the model is able to capture the fast response observed within a few months in clinical trials of NSCLC (32). Furthermore, the model was able to point towards less discussed characteristics of the responders in this virtual in silico clinical trial and made predictions about scenarios that were not explored clinically.

The primary strength of the model is in utilizing patient-specific parameters such as TMB and MHC/antigen affinity as input and to predict the likelihood of individual patients responding to anti-PD-1 therapy (nivolumab in this study). In this model, TMB was assumed to correlate with the number of clones of T cells that are activated, and MHC/antigen affinity was directly used in the antigen processing and presentation module that affects the efficiency of mAPC-mediated T cell activation. The model predicted that among the two parameters, TMB was the more important predictor of the response in the clinically relevant range. This prediction correlates with the published comprehensive analyses of anti-PD-1 therapy in NSCLC and SCLC clinical trial data (13,14). Additionally, model predicts that patients that undergo resection could benefit from adjuvant anti-PD-1 treatment in addition to neoadjuvant treatment. Although all the patients in Forde *et al.* (11) trial were diagnosed with stage I, II, or IIIA lung cancer who did not have detectable metastasis, these types of patients have lower than 50% 5-year survival rate and most cases have post-surgery tumor relapse (33). The clinical trial demonstrated that neoadjuvant treatment before the resection improves tumor regression, that is hoped to be translated to better overall survival in these patients. This model builds upon the clinical trial findings and predicts that addition of the adjuvant anti-PD-1 treatment could reduce the number of relapses in patients with high TMB by enhancing the killing capacity of Teff to eradicate any remaining metastases post-surgery. MHC/antigen affinity was another parameter that was quantified for the patients, but it did not correlate with the response for the majority of the patients, most likely because the median K_d_ only changed within an order of magnitude (12.4–88.3 nM). Only one patient had an expected negative response based on low MHC/antigen affinity, which was also the patient with the lowest TMB. Future implementation of a larger patient dataset in the model can help us to accurately tease out the contributions of these two factors. This model could be used as an input for virtual patient population generation algorithms (34–36) to enhance the power of model predictions.

Antigen processing and presentation is an important step in initiation of effective anti-tumor immune response, and detailed implementation of this feature revealed the dependence of response on abundance and clonality of antigenic and self-peptides. As discussed previously, TMB directly affects tumor size due to larger expansion of Teff that led to presence of more Teff in the tumor site to eradicate the tumor. Additionally, the model demonstrated that the MHC/antigen binding affinity plays an important role in effective activation of CD8 T cells by mAPCs in LN. At much higher K_d_ or much lower antigen availability (very small tumor), the number of presented antigens dropped dramatically, which resulted in inefficient activation of T cells even at high TMB. The model also added insight into the role of self-peptides in activation of Tregs and in turn diminishing of the Teff response at tumor site. Reduction in tumor size was often achieved in cases with efficient Teff response that lacked extensive Treg activation, which are primarily determined by features expressed by antigen processing and presentation module. Furthermore, the model can be expanded to explore polyclonal immune response to a tumor with antigens that have a range of MHC binding affinities.

In addition to TMB and antigen presentation-related parameters, the model identified a set of prior to therapy observables such as CD8 T cell clonality in blood or abundance of Teff and Treg and their ratio in the tumor, as well as parameters such as the density of naïve T cell in the blood, number of TdLNs, and T cell killing rate as important markers for higher chance of tumor shrinkage. Although we have not been able to readily validate the prediction of the model due to scarcity of available data in the literature, the future research aims to quantify the numbers of different cell types in the resected tumor samples from the patients using a validated multiplex immunofluorescence approach (25,37). One of the limitations of the current model is the assumption that naïve T cells of all TCR (T cell receptor) variations are always available in excess. Identification of the naïve T cell densities in blood as important parameters of the model suggests that future models need to represent the dynamics of the naïve T cells in the blood by implementing the thymic outputs for each simulated clone. CD8 T cell clonality could be measured by TCR-sequencing of the CD8 T cells in patient’s blood, although it is not trivial to identify the tumor-specific clones unless by in vitro examination of T cell expansion in response to patient antigen (28,38), or probabilistic estimation using sequence similarity of antigen to foreign epitopes identified in the Immune Epitope Database (IEDB) (39). T cell killing is not regarded as a parameter that can be targeted directly; however, approaches such as use of chimeric antigen receptor (CAR) T cells and bispecific T cell engagers (BiTE) could make it possible to modulate this parameter. Initial tumor size was another important parameter predicted to affect tumor diameter. Tumor burden has been shown to not significantly correlate with survival in recent clinical trials with nivolumab (13). The divergence might be due to the fact that our investigation is done at 1-year time point versus overall survival in actual patients. The unresponsive tumors with small initial diameter in the model would grow towards the maximum possible diameter, which in turn skews the results when we look at the correlation with percentage change in tumor diameter. In some of the cases, tumors with small diameter first grew to larger diameter at which the number of mAPC in the TdLN or the amount of antigen reached a large enough quantity to support a strong anti-tumor Teff activation. Number of APC in the tumor was assumed to correlate with tumor volume, and all mAPC were treated as they are able to migrate from tumor to the TdLN, which might not hold true in all the tumors either because of the unfavorable local chemokine gradients for APC entry and mAPC egress or limited lymphangiogenesis (40,41). Furthermore, this model only considers the tumor-associated neoantigens and not the self-antigens upregulated in the cancer cells such as the germline antigens (42–44), which could significantly contribute to the anti-tumor immune response.

Variation of dosing regimen parameters emphasized the necessity of continuation of biweekly dosing for effective tumor eradication. This is primarily because of dynamics between the cancer cell killing and immune activation. Nivolumab augments cancer cell death by inhibition of PD-1-mediated Teff exhaustion that pushes the cycle towards more Teff activation and proliferation and ultimately tumor elimination. Thus, continuous dosing in the whole period of 1 year is necessary to achieve a compounded anti-tumor immune response that could result in effective tumor size reduction. Increased dose amount and reduced interval between the doses for the limited range explored here did not significantly improve the result, but they would also likely increase the side effects of immune checkpoint blockades, most notably auto-immune-related complications (45). One of the reasons for potential discrepancy between the results of the model and clinical trials on dose exploration could be the fact that the virtual patients in this study are not fitted to the distribution of the clinical patient population. Elaborate virtual patient population generation algorithms could be added to this work based on the published studies on the virtual clinical trials (34–36). In a recent study, Basak *et al.* identified a longer overall survival rate in patients with higher trough concentration of nivolumab in a small cohort of NSCLC patients receiving nivolumab as the second-line treatment (46). These findings highlight the potential role of nivolumab exposure on the response, which was also suggested by this model. Further examination of this hypothesis in larger clinical trials is necessary for a definitive answer.

Our confidence in the model findings clearly depends on the accuracy of the experimental data used to constraint the model. Due to the scarce availability of the experimental data on anti-tumor immune response in human, there are inherent limitations in the predictive powers of the model. NSCLC is highly heterogenous both spatially and genetically, but as the first approximation, this study assumed that all the cancer cells in the tumor were homogenously distributed with uniform TMB. For purpose of the model simplification, we also assumed a polyclonal Teff response with identical clonal characteristics (e.g., MHC/antigen binding affinity and number of naïve T cell in each clone). It is assumed that the majority of the immune activation is orchestrated in the TdLN, although recent findings suggest important contribution of tertiary lymphoid organs (TLO) often formed just outside the tumor (25,47). Perhaps when comparing to experimental data, the total number of LNs in the model should be treated as the total number of TdLNs + TLOs, which would suggest that the presence of TLO should correlate with better response (Fig. 4b). Additionally, it was presumed that Teff could recognize cancer cells, which is an inherent limitation of the model and could be addressed in the future by implementing methods similar to the ones developed by Luksza *et al.* (39). To simplify the model at this stage, we neglected the role of IFNγ released by Teff in regulation of PD-L1 on cancer cells. Among the negative regulators of Teff activity in the tumor, Treg dynamics were included in the model. In the future studies, the dynamics of macrophages and myeloid-derived suppressor cells (MDSC) could be included in the model depending on the context of the study (48). Recent studies have explored the hypothesis that increased catabolic activity from anorexia/cachexia in patients could increase the clearance of antibody therapeutics and in turn indicate a correlation between the tumor burden and overall survival (49). In addition to the neoantigens modeled here, cancer germline antigens such as MAGE1, MAGE3, and NYESO1 are identified in various tumors (42–44) and could significantly contribute to the anti-tumor immune response. Implementation of these antigens in the future models would improve the predictive capabilities of the model and could explain the lack of correlation of response with TMB in some cancer types. The patient-specific pharmacokinetic parameters are often explored using the well-established population pharmacokinetic models, which potentially could be added to this model. An expected limitation of the model is the impossibility of global calibration of such a large model in the absence of equally extensive experimental data. Well-established parameter sensitivity analysis methods were utilized to ensure the identification of the important model parameters (23,35,50). In the future studies, combination of this QSP model with the agent-based models of tumor growth with immune cell infiltration would allow us to better understand the contribution of spatial localization of the Teff and Treg in patient response (16,51). Furthermore, with the increased attention to the role of immune response in control and elimination of cancer, our knowledge of anti-tumor immune response is constantly evolving either by identification of new mechanisms and/or enhanced understanding of the contribution of the already known mechanisms (52). Our model could be expanded or adapted to include any of these mechanisms depending on the specific tumor, particular therapy, or certain question that requires additional refinement of the model.

## Conclusions

In summary, by integrating our knowledge of anti-tumor immune response with detailed inclusion of antigen processing and presentation, we have built a comprehensive QSP model capable of explaining the modes of response based on patient characteristics. The model was calibrated based on the available clinical data on human NSCLC and was able to qualitatively reproduce the available experimental data. This model was utilized to explore the potential response in the patients from NCT02259621 trial that implemented neoadjuvant nivolumab therapy before surgical resection of the NSCLC tumors and showed the relative importance of TMB versus MHC/antigen binding affinity. With the expansion of the data collection in future clinical trials, including combination immunotherapies, this model can be further constrained for individual patients and patient cohorts using the information on tumor size and immune profiles in the blood and tumor samples to increase the patient-specific prediction power of the model.

## Electronic Supplementary Material

ESM 1 (DOCX 5106 kb)

ESM 2 (XLSX 32.8 kb)

ESM 3 (XML 244 kb)
