# Supplementary material for: A Computational Model of Neoadjuvant PD-1 Inhibition in Non-Small Cell Lung Cancer
Source: AAPS J. 2019 Jun 24;21(5):79. doi: 10.1208/s12248-019-0350-x (PMC6591205; doi:10.1208/s12248-019-0350-x)
Supplement: Supplementary file 1 — (DOCX 5106 kb) [file 12248_2019_350_MOESM1_ESM.docx]

# A Computational Model of Neoadjuvant PD-1 Inhibition in

# Non-Small Cell Lung Cancer

# Mohammad Jafarnejad, Chang Gong, Edward Gabrielson, Imke H. Bartelink, Paolo Vicini, Bing Wang, Rajesh Narwal, Lorin Roskos, Aleksander S. Popel

Supplementary Information

The details of all equations are available through the listed reactions, rules and events in the supplementary tables and can be directly explored through the provided model in SBML format. Additionally, compartments, species, parameters, are listed with their relevant values, units and explanation. For parameters the references to the literature sources used for parameter estimation are listed at the end of the notes. To help with understanding of different modules in the model, we explain the major components of the model here.

Tumor growth

Cancer growth was modeled using a well-established logistic model ([1](#_ENREF_1), [2](#_ENREF_2)) that considers the fast growth for the small diameter and saturation for large diameters due to physical and angiogenesis-related limitations. A first order death rate was assumed for basal death of cancer cells

$$\frac{\text{dC}}{\text{dt}}\text{=}{\text{ }\text{k}}_{\text{C,growth}}\text{C}\left( \text{1}-\frac{\text{C}}{\text{C}_{\text{max}}} \right)-\text{k}_{\text{C,death,base}}\text{C}-\text{ }\text{TKR}$$

where *C* is the number of cancer cells in the tumor, ${\text{ }\text{k}}_{\text{C,growth}}$ is the cancer cell growth rate, $\text{C}_{\text{max}}$ is maximum number of cancer cells that the tumor could reach, $\text{k}_{\text{C,death,base}}$ is basal cancer cell death rate*,* and *TKR* is Teff killing rate. *TKR* is expressed as an inverse Hill equation as follows

$$TKR =\text{ }k_{C,death,T_{eff}}\frac{C \cdot\text{ }T_{eff}}{C +\text{ }T_{tot}}\left( 1-\frac{{PD1\_PDLX}^{2}}{{PD1\_PDLX}^{2}+\text{ }K_{PD1\_PDLX}^{2}} \right)$$

where $\text{k}_{\text{C,death}\text{,}\text{T}_{\text{eff}}}$ is cancer killing rate by Teff, $\text{T}_{\text{eff}}$ is total Teff in the tumor, $\text{T}_{\text{tot}}$ is total T cells in the tumor, $PD1\_PDLX$ is the total number of engaged PD-1 molecules to PD-L1 and PD-L2 in the synapse, and $\text{K}_{\text{PD1\_PDLX}}$ is sensitivity of *TKR* to $PD1\_PDLX$. Binding of nivolumab to PD-1 will deplete the free PD-1 present on T cell that reduces the total $PD1\_PDLX$. This results in enhanced *TKR* due to the inverse Hill equation. Additionally, the Michaelis-Menten type rate that assumes the K_M_ constant to be dependent on the overall number of T cells in the compartment, which is also a measure of interaction volume ([3](#_ENREF_3), [4](#_ENREF_4)). This rate is used to reduce the model by combining the multiple steps of cell-cell interactions that results in an effect.

Antigen processing and presentation

The antigen processing and presentation is primarily modeled based on well-developed and published models of this process ([3](#_ENREF_3), [5-7](#_ENREF_5)). APC dynamics is modeled using a constant entry and first order entry exit/death. Maturation is modeled based on the availability of maturation signal ([3](#_ENREF_3)). The APC upon maturation (becoming mAPC) supposedly upregulate chemokine receptors such as CCR7 to migrate toward the lymphatic vessels and move to TdLN ([8](#_ENREF_8), [9](#_ENREF_9)). Antigenic and self-proteins are released from death of cancer cells and are internalized into intracellular vesicles and broken down into peptides. These peptides then bind MHC molecules depending on their binding affinity and are brought to the cell surface to be presented. Equations governing the uptake, processing and presentation of an antigen are

$$\frac{dP_{endo}}{dt}={k_{up} V}_{uptake}\frac{P_{tum}}{f_{tum}}-k_{deg,P}P_{endo}$$

$$\frac{dp_{endo}}{dt}=k_{deg,P}P_{endo}-k_{on}M_{endo}p_{endo}+k_{off}{M\text{\_}p}_{endo}-k_{deg,p}p_{endo}$$

$$\frac{d{M\_p}_{endo}}{dt}=k_{on}M_{endo}p_{endo}-k_{off}M\_p_{endo}-k_{deg,M\text{\_}p}M\_p_{endo}-k_{surf}M\_p_{endo}$$

$$\frac{d{M\_p}_{surf}}{dt}=k_{surf}M\_p_{endo}-k_{off}M\_p_{surf}$$

$$\frac{dM_{endo}}{dt}=k_{prod}M_{endo,0}-k_{deg,M}M_{endo}+k_{int}M_{surf}-k_{on}M_{endo}p_{endo}$$

$$\frac{dM_{surf}}{dt}=-k_{int}M_{surf}+k_{off}M\_p_{surf}$$

where *P* is the concentration of the protein, *p* is the concentration of the peptide, *M* is the MHC molecule, *M_p* is the antigen/MHC complexes, *k_up_* is protein uptake rate, *V_uptake_* is volume being internalized, *f_tum_* is porosity of the tumor, *k_deg,X_* is the degradation of various species, *k_on_* and *k_off_* are the binding and unbinding rates for antigen and MHC interaction, *k_surf_* rate of complex surfacing, *k_prod_* is the rate of MHC production, *k_int_* is the internalization rate of MHC molecules, *M_endo,0_* is the number of free MHC molecules and the subscript *tum* is for tumor, *endo* is for endoplasmic vesicles, and *surf* is for APC surface membrane. Duplicates of these equations are used to simulate the processing and presentation of self-proteins. The overall degradation of the proteins in the extracellular space is assumed to be significantly higher than the uptake rate by APC/mAPC. To simplify the model, we assumed that the number of each peptide presented per APC/mAPC remains dependent on the tumor concentration of the proteins during the migration of mAPC to TdLN and until the cell death. Additionally, to reduce the number of equations, we assumed that APC and mAPC have similar processing and presentation mechanisms and the only difference is in the costimulation molecules that define mAPC activations of naïve T cells to effector cells and APC-mediated induction of Treg ([10](#_ENREF_10)).

T cell activation and proliferation

T cells activation in TdLN is based on a two-step priming model partially based on the previous works on this topic ([3](#_ENREF_3), [11](#_ENREF_11), [12](#_ENREF_12)). In first step, mAPC activate naïve T cells that depends on the number of antigenic peptides being presented on the MHC molecules:

$$\text{ }\text{TCAR}\text{ = }\text{k}_{\text{nTCD8,mAPC}} \cdot\text{n}_{\text{Ag,clone}}\left( \frac{\text{T}_{\text{naive,CD8}}}{\text{n}_{\text{TCD8,diversity}}} \right)\cdot\text{ }\left( \frac{\text{n}_{\text{site,mAPC}}\text{ }\cdot\text{ }\text{mAPC}}{\text{n}_{\text{site,mAPC}}\text{ }\cdot\text{ }\text{mAPC}\text{ }\text{+ }\frac{\text{T}_{\text{naive,CD8}}}{\text{n}_{\text{TCD8,diversity}}}} \right)\cdot\left( \frac{\frac{\text{n}_{\text{presented,Ag}}}{\text{n}_{\text{Ag,clone}}}}{\frac{\text{n}_{\text{presented,Ag}}}{\text{n}_{\text{Ag,clone}}}\text{ + }\text{K}_{\text{presentation}}} \right)$$

where *TCAR* is T cell activation rate, $\text{k}_{\text{nTCD8,mAPC}}$ is maximum activation rate of naïve CD8 T cells by mAPC, $\text{T}_{\text{naive,CD8}}$ is number of naïve CD8 T cells with any type of T cell receptor (TCR), $\text{n}_{\text{TCD8,diversity}}$ is number of types of CD8 T cell in the patient, $\text{n}_{\text{site,mAPC}}$ is average number of sites on mAPC for antigen presentation, *mAPC* is the number of mAPC in the TdLN, $\text{n}_{\text{presented,Ag}}$ is number of antigenic peptides presented on the mAPC, $\text{n}_{\text{Ag,clone}}$ is the number of antigenic peptide clones associated with the tumor, and $\text{K}_{\text{presentation}}$ is sensitivity of the *TCAR* to number of antigens presented on mAPC.

In second step, activated T cells proliferate into functional effector T cell expressed by:

$$\text{ }\text{TCPR}\text{ = }\frac{\text{k}_{\text{aTCD8,prolif}}}{\text{n}_{\text{prolif}}}\cdot\text{2}^{\text{n}_{\text{prolif}}}\cdot\text{T}_{\text{activated,CD8}}$$

where *TCPR* is T cell proliferation rate, $\text{k}_{\text{aTCD}\text{8,prolif}}$ is doubling rate of activated T cells, $\text{n}_{\text{prolif}}$ is the number of generations T cells proliferate, and $\text{T}_{\text{activated,CD8}}$ is the number of activated T cells in the TdLN. The number of generations that activated T cells proliferate (division destiny) before leaving the TdLN depends on TCR engagement, costimulation signal through CD28, and IL-2 receptor stimulation ([13](#_ENREF_13)). Dynamics of $\text{T}_{\text{activated,CD}\text{8}}$ and fully differentiated and proliferate effector CD8 T cells ($T_{eff}$) were represented explicitly. This derivation is based on the work by Jones and Perelson ([14](#_ENREF_14)) and assumes pseudo-equilibrium for the intermediate proliferating states as well as a first proliferation step that inversely depends on division destiny ([15](#_ENREF_15)). Furthermore, because only the fully differentiated and proliferated T cells were of interest in the model, the proliferating cells in the intermediate steps were no modeled explicitly. One of the reasons for further simplifying the equations from Jones and Perelson ([14](#_ENREF_14)) was that division destiny in this model was a variable positive real number, and not an integer. The data from Marchingo et al. ([13](#_ENREF_13)) was used to find an expression for division destiny, which depends on the IL-2 dynamics in the TdLN. IL-2 release is assumed to be dependent on the number of activated T cells, and IL-2 degradation depended on specific affinity of IL-2 binding to heterodimeric IL-2R on CD8 T cell and higher affinity of IL-2 binding to heterotrimeric IL-2R on Treg ([13](#_ENREF_13), [16](#_ENREF_16)). This model assumes that the density of the naïve T cells in blood is constant and does not consider possible depletion of naïve T cells of the reactive clones. One of the limitations of this model is the assumption that all the antigens result in activation of a T cell clone. Overall, this assumption overestimates the anti-tumor T cell clones because there would be antigens that lack appropriate T cell clones in the patient. The model developed by Luksza et al. had the advantage of comparing the antigen sequence with the Immune Epitope Database (IEDB) to estimate the probability of reactive T cell clone presence in the patients ([17](#_ENREF_17)).

T cell distribution

The T cell distribution in this study is based on the model by Zhu et al ([18](#_ENREF_18)). Terminally proliferated Teff egress the TdLN to blood. Teff in the central compartment are distributed to tumor and peripheral compartments. We have assumed that Teff cells probe peripheral tissues and leave, but they stay in the tumor because of the chemokine gradients until they die. Additionally, Treg and cancer cells can induce Teff exhaustion in the tumor. T cell clonality in the blood is defined as 1-Pielou’s evenness ([19](#_ENREF_19)) using the following equation:

$$\text{Clonality = 1+}\frac{\sum_{\text{i}}^{\text{N}} \text{p}_{\text{i}}\text{log}_{\text{2}}\text{(}\text{p}_{\text{i}}\text{)}}{\text{log}_{\text{2}}\text{(}\text{N}\text{)}}$$

where $p_{i}$ is the probability or population fraction of *i*th type of CD8 T cell in the blood, and $N$ is the total number of types of T cells in the blood (expanded clones plus naïve CD8 T cells).

Treg dynamics

Induction of Treg is assumed to follow similar equations to CD8 T cell activation, except that the number of self-peptides on the APC (but not mAPC) activates CD4 T cells to become Treg ([10](#_ENREF_10), [20](#_ENREF_20)), and the number of proliferation generations only depends on IL-2 mechanisms ([16](#_ENREF_16)). The distribution of Treg follows equations similar to the Teff. In addition to the induced Treg, natural Treg are modeled by a release rate from thymus that results in a baseline level which was observed experimentally ([21](#_ENREF_21)). Furthermore, Treg in tumor are assumed to inhibit Teff by two mechanisms. First, PD-1/PD-L1 mediated mechanisms that account for a third of the maximum inhibition, and second, other mechanisms such as release of cytokines (e.g. IL-10) ([22](#_ENREF_22)).

Immune checkpoint blockade dynamics

PD-1 on Teff interacts with PD-L1 and PD-L2 on cancer cell or Treg in the immunological synapse that causes reduced cancer killing by Teff, Teff exhaustion, and partial reduction in Treg inhibition of the Teff. The interactions in PD-1/PD-L1/PD-L2 axis have been characterized and modeled before ([23](#_ENREF_23)), and a simplified version of this model was adapted for this study. Briefly, we assumed the abundance of the ligands and receptors in the immunological synapse is proportional to the surface area of the synapse compared to the total cell surface area. In this model, we assumed the fast dynamics from explicit representation of the diffusive entry of surface molecules to the synapse has negligible effect and in turn represented the effect of diffusion by overestimating the area of the synapse by a factor of 3. This was achieved by comparing the model used in this study with the previously published models that explicitly implemented the diffusion ([7](#_ENREF_7), [23](#_ENREF_23)). The numbers of checkpoint molecules on cells were estimated based on measurements using quantitative flow cytometry using calibrated fluorescent beads ([23](#_ENREF_23), [24](#_ENREF_24)). We used PD-1 measurements from Mkrtichyan et al. ([24](#_ENREF_24)) and scaled up the rest of the measurements from Cheng et al. ([23](#_ENREF_23)) to account for possible underestimation of PD-L1 and PD-L2 from QuantiBRITE bead measurements. These parameters are varied in a wide range in parameter sensitivity analysis to investigate the uncertainty in the measurements. The antibody binding to the PD-1 was modeled using a bivalent model of antibody receptor interaction on cell surface ([25](#_ENREF_25), [26](#_ENREF_26)). Thus, the governing equations for the dynamics of the checkpoint molecules in the immune synapse are:

$$\frac{dPD1\_PDL1}{dt}=k_{on,PD1\_PDL1}\cdot PD1\cdot PDL1-k_{off,PD1\_PDL1}\cdot PD1\_PDL1$$

$$\frac{dPD1\_PDL2}{dt}=k_{on,PD1\_PDL2}\cdot PD1\cdot PDL2-k_{off,PD1\_PDL2}\cdot PD1\_PDL2$$

$$\frac{dPD1\_Nivo}{dt}=2k_{on,PD1\_Nivo}\cdot PD1\cdot Nivo/f_{tum}-k_{off,PD1\_Nivo}\cdot PD1\_Nivo$$

$$\frac{dPD1\_Nivo\_PD1}{dt}=X\left( \frac{k_{on,PD1\_Nivo}}{A_{syn}d_{syn}N_{A}} \right)\cdot PD1\_Nivo\cdot PD1-{2k}_{off,PD1\_Nivo}\cdot PD1\_Nivo\_PD1$$

$$\frac{dPD1}{dt}={-k}_{on,PD1\_PDL1}\cdot PD1\cdot PDL1+k_{off,PD1\text{\_}PDL1}\cdot PD1_{PDL1}-k_{on,PD1\text{\_}PDL2}\cdot PD1\cdot PDL2+k_{off,PD1\text{\_}PDL2}\cdot PD1\text{\_}PDL2-{2k}_{on,PD1\text{\_}Nivo}\cdot PD1\cdot\frac{Nivo}{f_{tum}}+k_{off,PD1\text{\_}Nivo}\cdot PD1\text{\_}Nivo-{Xk}_{on,PD1\_Nivo}\cdot PD1\_Nivo\cdot PD1-{2k}_{off,PD1\_Nivo}\cdot PD1\_Nivo\_PD1$$

where $k_{on,PD1\_X}$ and $k_{off,PD1\_X}$ are the on and off rates for interactions between PD-1 and X (PD-L1, PD-L2, and nivolumab), $f_{tum}$ is the porosity in the tumor, $X$ is the intrinsic antibody cross-arm binding efficiency, $A_{syn}$ is surface area of the synapse, $d_{syn}$ is the thickness of the confinement space between the two cells, and $N_{A}$ is Avogadro’s number. Because of the different units for $Nivo (nM)$ versus $PD1\_Nivo (molecule)$, $k_{on,PD1\_Nivo}$ was converted to units of $1/{(molecule.s)}$ using the synapse sizes and Avogadro’s number. The number of bound PD-1 molecules on Teff was translated to Teff exhaustion using a Hill equation. PD-1/PD-L1/PD-L2 dynamics is assumed to be similar for Teff and Treg, thus only a single checkpoint module was used in this study to reduce the size of the model in the absence of the data. The baseline parameters for module was chosen to fit Hill function output to *in vitro* nivolumab dose-response measurements of IFNγ (Figure S7)([27](#_ENREF_27)). Effect of PD1 ligation on T cell exhaustion was included in two reactions of Treg and cancer cell-mediated inhibition of Teff.

Antibody pharmacokinetic

Pharmacokinetic of anti-PD-1 antibody, nivolumab, was modelled using a physiologically-based pharmacokinetic model previously described in detail ([28](#_ENREF_28)). Briefly, antibody dose was infused with a constant rate over a period of 30 min (as performed clinically for nivolumab) and a first order clearance from the blood was implemented. The antibody transport from the blood to other compartments (i.e. tumor, TdLN, and peripheral) followed transvascular diffusion of the antibody across the blood microvessels ([29](#_ENREF_29)). Additionally, the lymphatic-mediated transport of antibody from tumor to TdLN was described by a simple convection term that depends on the lymphatic drainage of the solid tumors based on Zhu et al. ([18](#_ENREF_18)) and the concentration of the antibody in the tumor. The equations governing the antibody pharmacokinetics are as follows:

$$V_{C}\frac{dA_{C}}{dt}=q_{P}\left( A_{P}-A_{C} \right)+q_{LN}\left( A_{LN}-A_{C} \right)+q_{T}\left( A_{T}-A_{C} \right)+q_{LD}A_{LN}-k_{cl}$$

$$V_{P}\frac{dA_{P}}{dt}=q_{P}\left( A_{C}-A_{P} \right)$$

$$V_{T}\frac{dA_{T}}{dt}=q_{T}\left( A_{C}-A_{T} \right)+q_{LD}A_{T}$$

$$V_{LN}\frac{dA_{LN}}{dt}=q_{LN}\left( A_{C}-A_{LN} \right)+q_{LD}A_{T}-q_{LD}A_{LN}$$

In addition to complete agreement with the clinical measurements of the antibody concentration dynamics in the plasma, this physiologically-based model predicted an antibody biodistribution coefficient of 22.7% for the NSCLC tumor that is expected to be higher than the healthy lung (14.9%) due to the leakier vessels in the growing tumor ([30](#_ENREF_30)).

Supplementary Tables

Table S1. Parameters, geometric mean and standard deviation in which they were varied for the parameter sensitivity analysis.

| Parameter | Geometric mean | Geometric standard deviation | Unit |
| --- | --- | --- | --- |
| Initial Tumor Volume | 0.07 | 1 | liter |
| Tumor Growth Rate | 0.01 | 2 | 1/day |
| Basal Tumor Death Rate | 0.0001 | 2 | 1/day |
| Rate of Tumor Death by Teff | 8 | 2 | 1/day |
| Rate of Treg Inhibition of Teff | 1 | 2 | 1/day |
| Number of Ag Clones (TMB) | 92 | 2 | dimensionless |
| K_D_ of Ag-MHC | 40 | 2 | nanomolarity |
| Sensitivity of PD-1 inhibition of Teff | 250 | 2 | molecule |
| Total PD-1 molecules on Teff | 60000 | 2 | molecule |
| Total PD-L1 molecules on cancer cells | 1600000 | 2 | molecule |
| Total PD-L2 molecules on cancer cells | 104000 | 2 | molecule |
| T cell inactivation by Cancer cells | 0.05 | 2 | 1/day |
| Number of LNs | 21 | 2 | dimensionless |
| K_D_ of self-peptides | 60 | 2 | nanomolarity |
| Rate of naive T cell entry to LN | 0.07 | 2 | 1/day/milliliter |
| Ag-MHC for half-maximal T cell activation | 400 | 2 | molecule |
| Density of naive CD8 T cell in blood | 505000 | 1 | cell/milliliter |
| Density of naive CD4 T cell in blood | 860000 | 1 | cell/milliliter |
| Adhesion site density in tumor | 100000000 | 2 | cell/(centimeter^3) |
| Blood vessel fraction in tumor | 0.0067 | 2 | dimensionless |
| Maximum Tumor Volume | 1 | 2 | liter |
| Steady state density of APC in tumor | 400000 | 2 | cell/milliliter |
| Steady state density of APC in LN | 1200000 | 2 | cell/milliliter |
| Number of Treg clones | 250 | 2 | dimensionless |
| K_D_ of PD1 and PD-L1 interaction | 0.097 | 1 | Molecule/ (micrometer^2) |
| K_D_ of PD1 and PD-L2 interaction | 4.2 | 1 | Molecule/ (micrometer^2) |
| Hill coefficient of PD1-PDLX | 2 | 1 | dimensionless |
| Surface area of the synapse | 37.8 | 1 | micrometer^2 |
| K_D_ of PD-1 and single arm nivolumab binding | 0.26 | 1 | nanomolarity |
| Clearance of the nivolumab | 0.062 | 0.1 | 1/day |

Table S2. Patient-specific parameters used in this study based on measured values in the clinical trial ([31](#_ENREF_31)).

|  | Patient ID | TMB  (No. Sequence Alterations) | Median MHC/antigen affinity (nM) |
| --- | --- | --- | --- |
| Patient 01 | MD01-005 | 256 | 52.8 |
| Patient 02 | MD01-004 | 99 | 29.4 |
| Patient 03 | MD043-008 | 310 | 29.2 |
| Patient 04 | MD043-012 | 68 | 17.4 |
| Patient 05 | MD043-011 | 75 | 84.9 |
| Patient 06 | MD01-019 | 105 | 30.4 |
| Patient 07 | MD043-006 | 190 | 25.0 |
| Patient 08 | MD01-024 | 23 | 41.4 |
| Patient 09 | NY016-007 | 5 | 733.0 |
| Patient 10 | NY016-014 | 26 | 12.4 |
| Patient 11 | NY016-016 | 366 | 88.3 |
| Patient 12 | NY016-017 | 85 | 59.4 |

Supplementary Figure Captions

Figure S1. p-value for PRCC in Figure 4.

Figure S2. Patient response under no treatment. TMB and MHC/antigen affinity were set according to the measured patient data and the rest of 30 parameters were randomly changed based on Latin hypercube sampling for 200 simulations/patient under no treatment. The data are reported as median ± 30%, 60% and 90% prediction interval.

Figure S3. Predicted patient response under biweekly nivolumab treatment. TMB and MHC/antigen affinity were set according to the measured patient data and the rest of 30 parameters were randomly changed based on Latin hypercube sampling for 200 simulations/patient under biweekly nivolumab treatment. The data are reported as median ± 30%, 60% and 90% prediction interval.

Figure S4. Predicted patient response under neoadjuvant nivolumab plus resection. TMB and MHC/antigen affinity were set according to the measured patient data and the rest of 30 parameters were randomly changed based on Latin hypercube sampling for 200 simulations/patient under neoadjuvant nivolumab plus resection. The data are reported as median ± 30%, 60% and 90% prediction interval.

Figure S5. Effect of dosing regimen on the response was explored by varying dose number, amount and interval.

Simulations performed by selecting three conditions for dosing regimen parameters such as number of doses (red), amount of the drug (blue), and interval between administration (green) and under each set condition running 200 simulations with variation of 30 parameters described in parameter sensitivity analysis (Figure 4).

Figure S6. Effect of dosing regimen on the 1 year end point tumor size was explored by varying dose number, amount and interval.

Boxplots of endpoint tumor sizes while varying the three dosing parameters show median and interquartile range. None of the variations resulted in statistically significant changes. Histograms of tumor size at 1 year are shown by selecting three conditions for dosing regimen parameters such as number of doses (red), amount of the drug (blue), and interval between administration (green) and under each set condition running 200 simulations with variation of 30 parameters described in parameter sensitivity analysis (Figure 4).

Figure S7. Comparison of effect of bivalent antibody model parameters in nivolumab dose response.

Unknown parameters affecting the antibody-mediated inhibition of the PD-1 interactions are varied to identify a reasonable assumption supported by the experimental data from Wang et al ([27](#_ENREF_27)). Three parameters of intrinsic antibody cross-arm binding efficiency ($X$), half-maximal activation and Hill coefficient of the Hill equation are varied. The yellow traces show the baseline case used in the model.

References

1. Sarapata EA, DePillis LG. A comparison and catalog of intrinsic tumor growth models. Bulletin of mathematical biology. 2014;76(8):2010-24.

2. DePillis LG, Gallegos A, Radunskaya EA. A model of dendritic cell therapy for melanoma. Front Oncol. 2013;3:56.

3. Chen X, Hickling TP, Vicini P. A mechanistic, multiscale mathematical model of immunogenicity for therapeutic proteins: part 1-theoretical model. CPT Pharmacometrics Syst Pharmacol. 2014;3:e133.

4. Carneiro J, Coutinho A, Stewart J. A model of the immune network with B-T cell co-operation. II--The simulation of ontogenesis. J Theor Biol. 1996;182(4):531-47.

5. Chen X, Hickling TP, Vicini P. A mechanistic, multiscale mathematical model of immunogenicity for therapeutic proteins: part 2-model applications. CPT Pharmacometrics Syst Pharmacol. 2014;3:e134.

6. Palsson S, Hickling TP, Bradshaw-Pierce EL, Zager M, Jooss K, O'Brien PJ, et al. The development of a fully-integrated immune response model (FIRM) simulator of the immune response through integration of multiple subset models. BMC Syst Biol. 2013;7:95.

7. Agrawal NG, Linderman JJ. Mathematical modeling of helper T lymphocyte/antigen-presenting cell interactions: analysis of methods for modifying antigen processing and presentation. J Theor Biol. 1996;182(4):487-504.

8. Russo E, Teijeira A, Vaahtomeri K, Willrodt AH, Bloch JS, Nitschke M, et al. Intralymphatic CCL21 Promotes Tissue Egress of Dendritic Cells through Afferent Lymphatic Vessels. Cell reports. 2016;14(7):1723-34.

9. Weber M, Hauschild R, Schwarz J, Moussion C, de Vries I, Legler DF, et al. Interstitial dendritic cell guidance by haptotactic chemokine gradients. Science (New York, NY). 2013;339(6117):328-32.

10. Fouchet D, Regoes R. A population dynamics analysis of the interaction between adaptive regulatory T cells and antigen presenting cells. PLoS One. 2008;3(5):e2306.

11. De Boer RJ, Perelson AS. Towards a general function describing T cell proliferation. J Theor Biol. 1995;175(4):567-76.

12. De Boer RJ, Perelson AS. T cell repertoires and competitive exclusion. J Theor Biol. 1994;169(4):375-90.

13. Marchingo JM, Kan A, Sutherland RM, Duffy KR, Wellard CJ, Belz GT, et al. T cell signaling. Antigen affinity, costimulation, and cytokine inputs sum linearly to amplify T cell expansion. Science (New York, NY). 2014;346(6213):1123-7.

14. Jones LE, Perelson AS. Opportunistic infection as a cause of transient viremia in chronically infected HIV patients under treatment with HAART. Bulletin of mathematical biology. 2005;67(6):1227-51.

15. Gett AV, Hodgkin PD. A cellular calculus for signal integration by T cells. Nature immunology. 2000;1(3):239-44.

16. Wang HM, Smith KA. The interleukin 2 receptor. Functional consequences of its bimolecular structure. J Exp Med. 1987;166(4):1055-69.

17. Luksza M, Riaz N, Makarov V, Balachandran VP, Hellmann MD, Solovyov A, et al. A neoantigen fitness model predicts tumour response to checkpoint blockade immunotherapy. Nature. 2017;551(7681):517-20.

18. Zhu H, Melder RJ, Baxter LT, Jain RK. Physiologically based kinetic model of effector cell biodistribution in mammals: implications for adoptive immunotherapy. Cancer Res. 1996;56(16):3771-81.

19. Lavin Y, Kobayashi S, Leader A, Amir ED, Elefant N, Bigenwald C, et al. Innate Immune Landscape in Early Lung Adenocarcinoma by Paired Single-Cell Analyses. Cell. 2017;169(4):750-65.e17.

20. Alonso R, Flament H, Lemoine S, Sedlik C, Bottasso E, Peguillet I, et al. Induction of anergic or regulatory tumor-specific CD4(+) T cells in the tumor-draining lymph node. Nat Commun. 2018;9(1):2113.

21. Churlaud G, Pitoiset F, Jebbawi F, Lorenzon R, Bellier B, Rosenzwajg M, et al. Human and Mouse CD8(+)CD25(+)FOXP3(+) Regulatory T Cells at Steady State and during Interleukin-2 Therapy. Frontiers in immunology. 2015;6:171.

22. Zappasodi R, Budhu S, Hellmann MD, Postow MA, Senbabaoglu Y, Manne S, et al. Non-conventional Inhibitory CD4(+)Foxp3(-)PD-1(hi) T Cells as a Biomarker of Immune Checkpoint Blockade Activity. Cancer Cell. 2018;33(6):1017-32.e7.

23. Cheng X, Veverka V, Radhakrishnan A, Waters LC, Muskett FW, Morgan SH, et al. Structure and interactions of the human programmed cell death 1 receptor. The Journal of biological chemistry. 2013;288(17):11771-85.

24. Mkrtichyan M, Najjar YG, Raulfs EC, Liu L, Langerman S, Guittard G, et al. B7-DC-Ig enhances vaccine effect by a novel mechanism dependent on PD-1 expression level on T cell subsets. Journal of immunology (Baltimore, Md : 1950). 2012;189(5):2338-47.

25. Harms BD, Kearns JD, Su SV, Kohli N, Nielsen UB, Schoeberl B. Optimizing properties of antireceptor antibodies using kinetic computational models and experiments. Methods Enzymol. 2012;502:67-87.

26. Harms BD, Kearns JD, Iadevaia S, Lugovskoy AA. Understanding the role of cross-arm binding efficiency in the activity of monoclonal and multispecific therapeutic antibodies. Methods (San Diego, Calif). 2014;65(1):95-104.

27. Wang C, Thudium KB, Han M, Wang XT, Huang H, Feingersh D, et al. In vitro characterization of the anti-PD-1 antibody nivolumab, BMS-936558, and in vivo toxicology in non-human primates. Cancer immunology research. 2014;2(9):846-56.

28. Finley SD, Angelikopoulos P, Koumoutsakos P, Popel AS. Pharmacokinetics of Anti-VEGF Agent Aflibercept in Cancer Predicted by Data-Driven, Molecular-Detailed Model. CPT Pharmacometrics Syst Pharmacol. 2015;4(11):641-9.

29. Schmidt MM, Wittrup KD. A modeling analysis of the effects of molecular size and binding affinity on tumor targeting. Molecular cancer therapeutics. 2009;8(10):2861-71.

30. Shah DK, Betts AM. Antibody biodistribution coefficients: inferring tissue concentrations of monoclonal antibodies based on the plasma concentrations in several preclinical species and human. mAbs. 2013;5(2):297-305.

31. Forde PM, Chaft JE, Smith KN, Anagnostou V, Cottrell TR, Hellmann MD, et al. Neoadjuvant PD-1 Blockade in Resectable Lung Cancer. The New England journal of medicine. 2018;378(21):1976-86.

Supplementary Figures


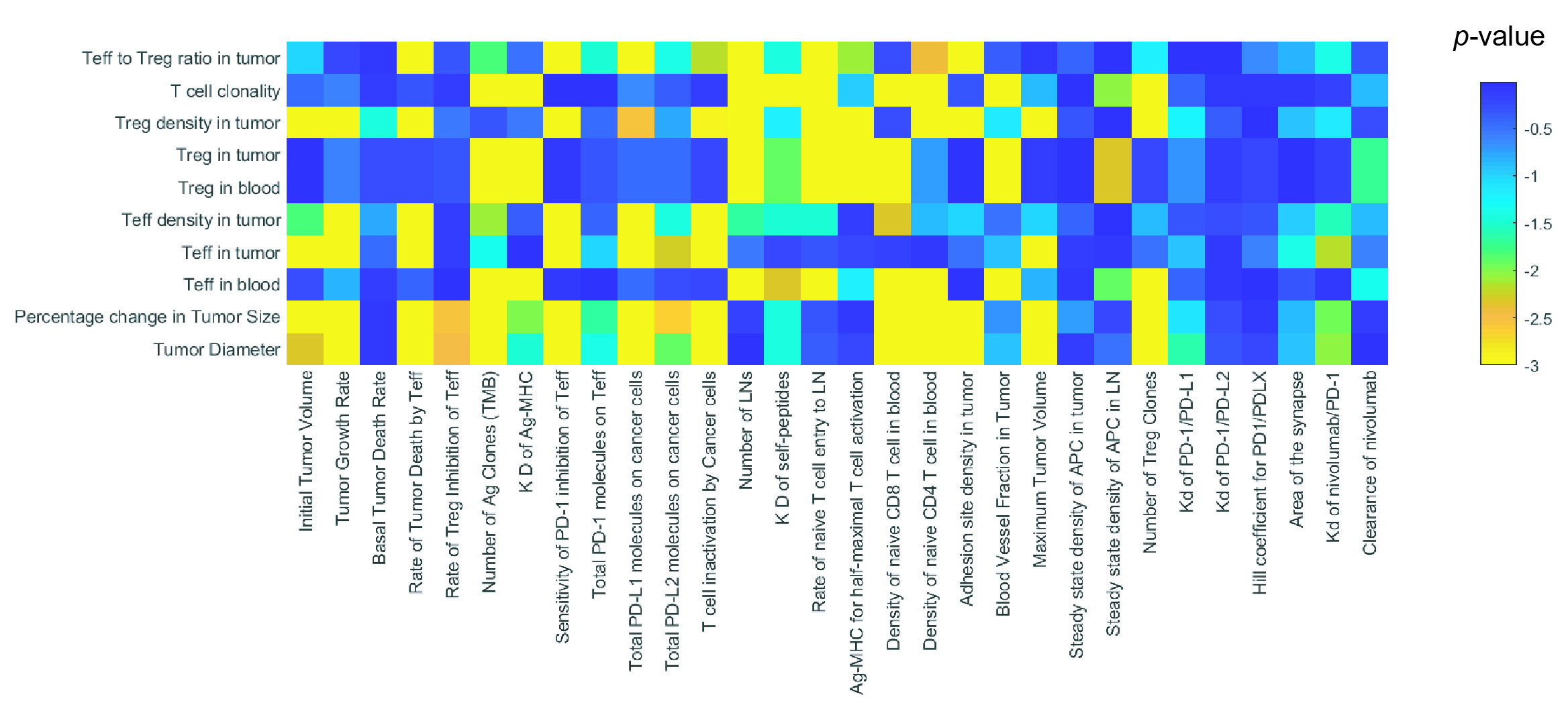


Figure S1. *p*-value for PRCC in Figure 4.


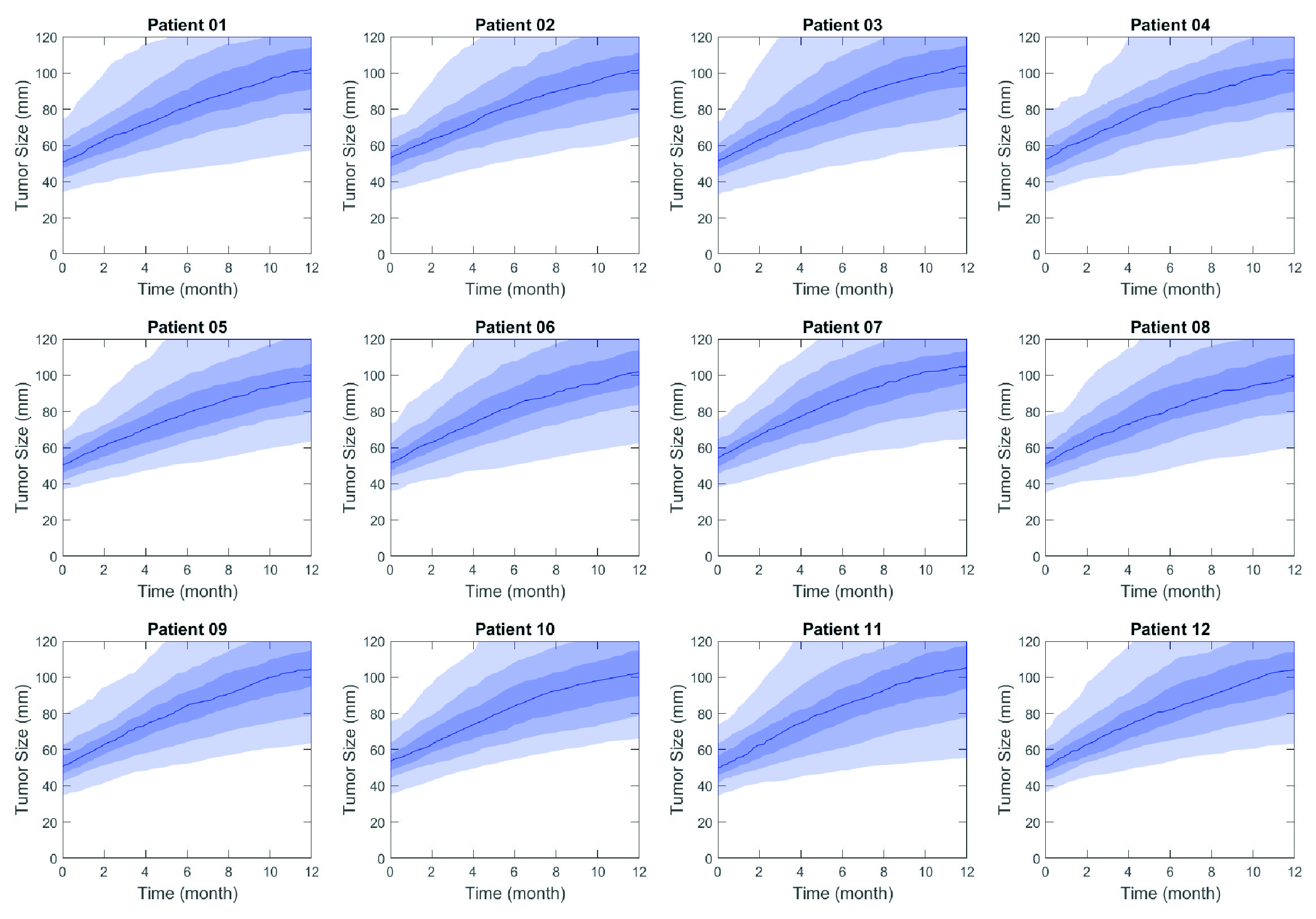


Figure S2. Patient response under no treatment. TMB and MHC/antigen affinity were set according to the measured patient data and the rest of 30 parameters were randomly changed based on Latin hypercube sampling for 200 simulations/patient under no treatment. The data are reported as median ± 30%, 60% and 90% prediction interval.


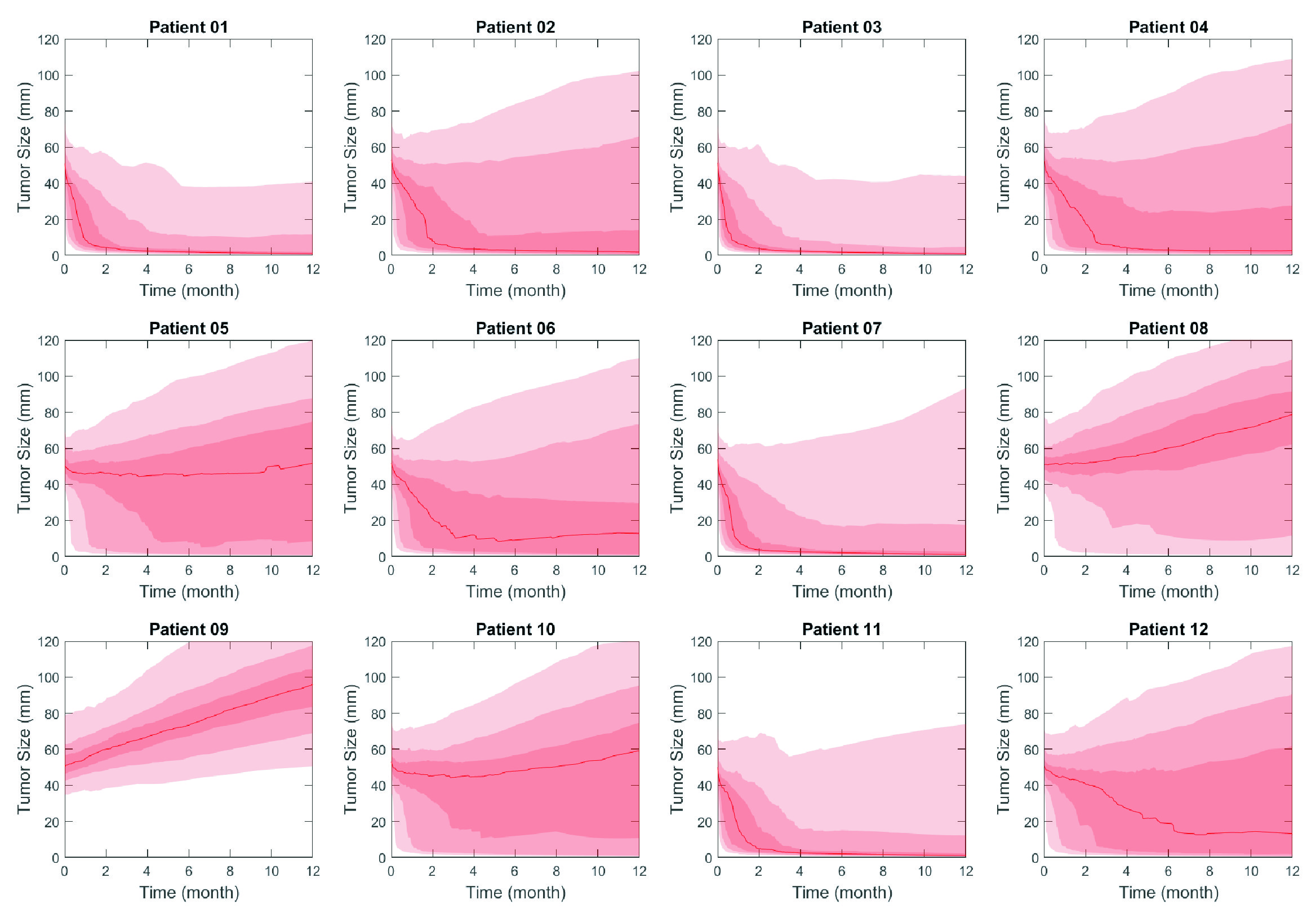


Figure S3. Patient response under biweekly nivolumab treatment. TMB and MHC/antigen affinity were set according to the measured patient data and the rest of 30 parameters were randomly changed based on Latin hypercube sampling for 200 simulations/patient under biweekly nivolumab treatment. The data are reported as median ± 30%, 60% and 90% prediction interval.


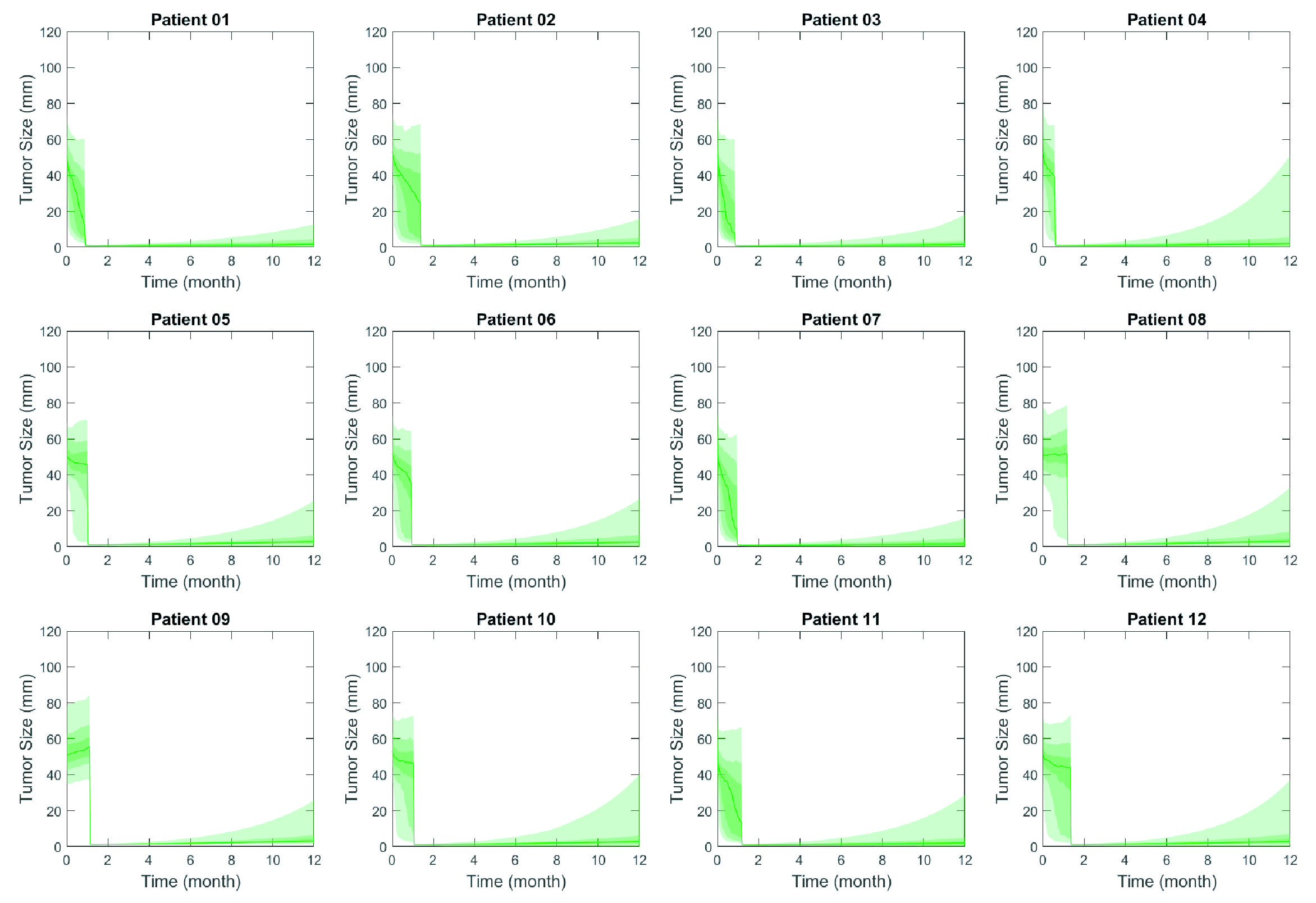


Figure S4. Patient response under neoadjuvant nivolumab plus resection. TMB and MHC/antigen affinity were set according to the measured patient data and the rest of 30 parameters were randomly changed based on Latin hypercube sampling for 200 simulations/patient under neoadjuvant nivolumab plus resection. The data are reported as median ± 30%, 60% and 90% prediction interval.


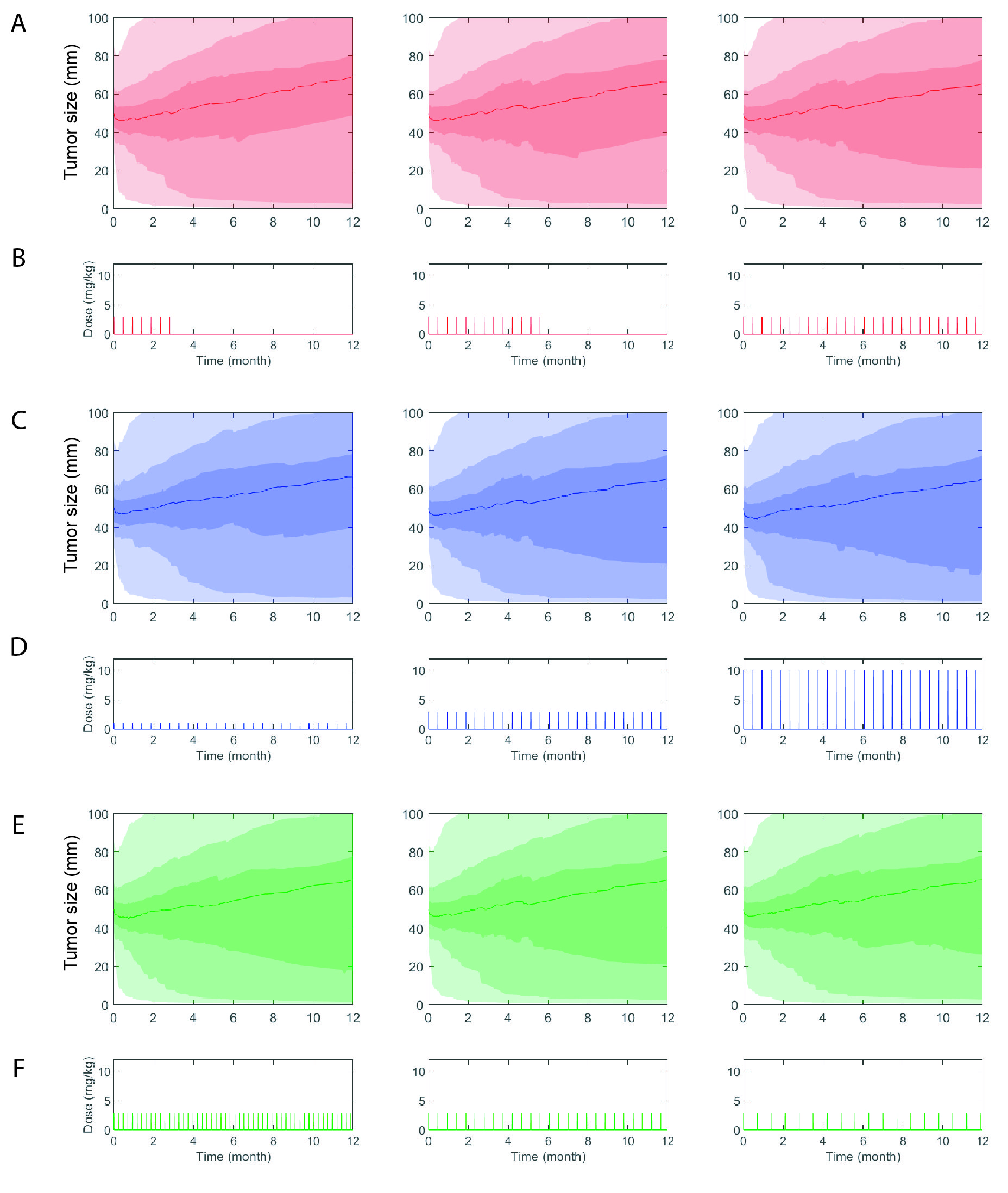


Figure S5. Effect of dosing regimen on the response was explored by varying dose number, amount and interval.

Simulations performed by selecting three conditions for dosing regimen parameters such as number of doses (red), amount of the drug (blue), and interval between administration (green) and under each set condition running 200 simulations with variation of 30 parameters described in parameter sensitivity analysis (Figure 4).


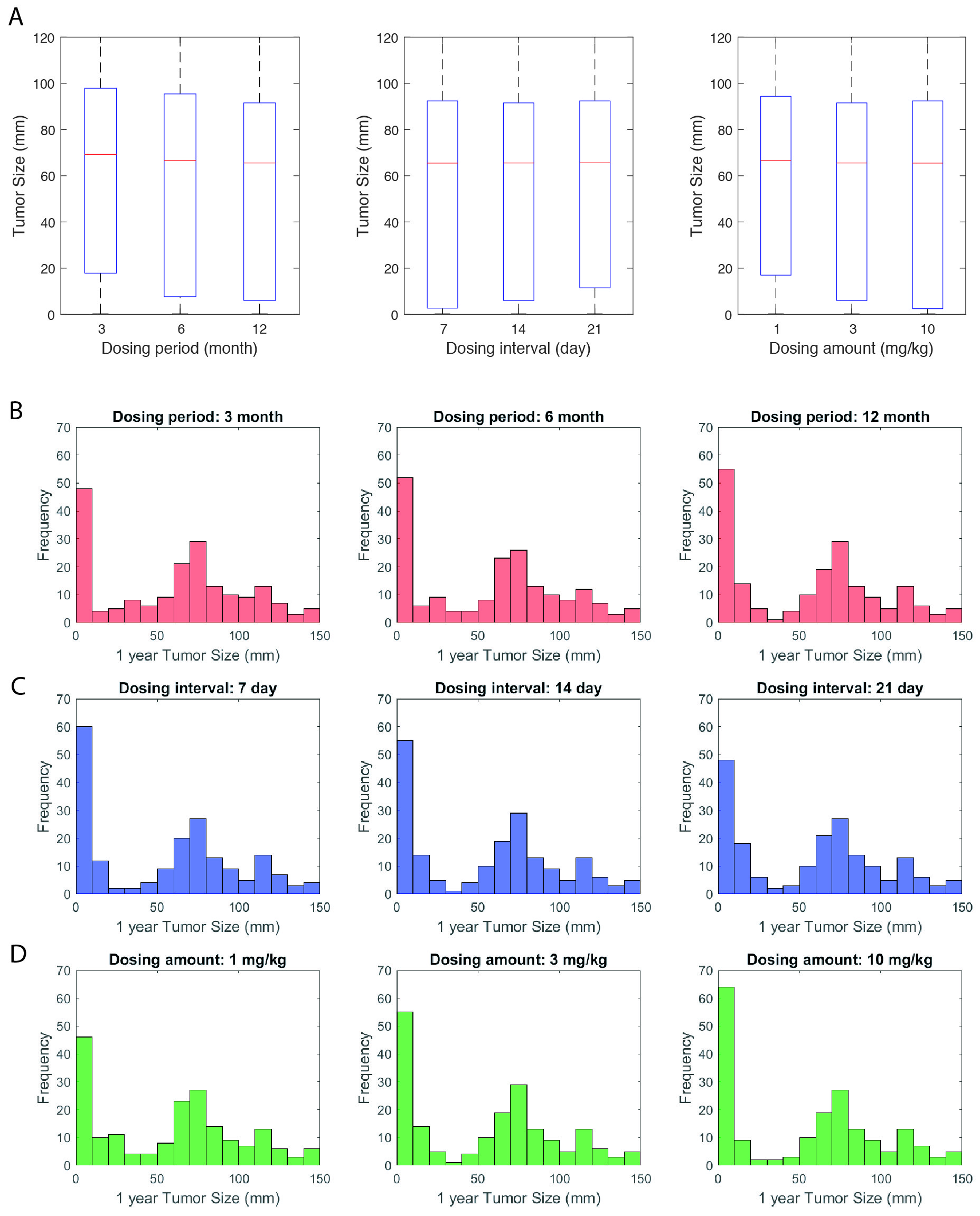


Figure S6. Effect of dosing regimen on the 1 year end point tumor size was explored by varying dose number, amount and interval.

Boxplots of endpoint tumor sizes while varying the three dosing parameters show median and interquartile range. None of the variations resulted in statistically significant changes. Histograms of tumor size at 1 year are shown by selecting three conditions for dosing regimen parameters such as number of doses (red), amount of the drug (blue), and interval between administration (green) and under each set condition running 200 simulations with variation of 30 parameters described in parameter sensitivity analysis (Figure 4).


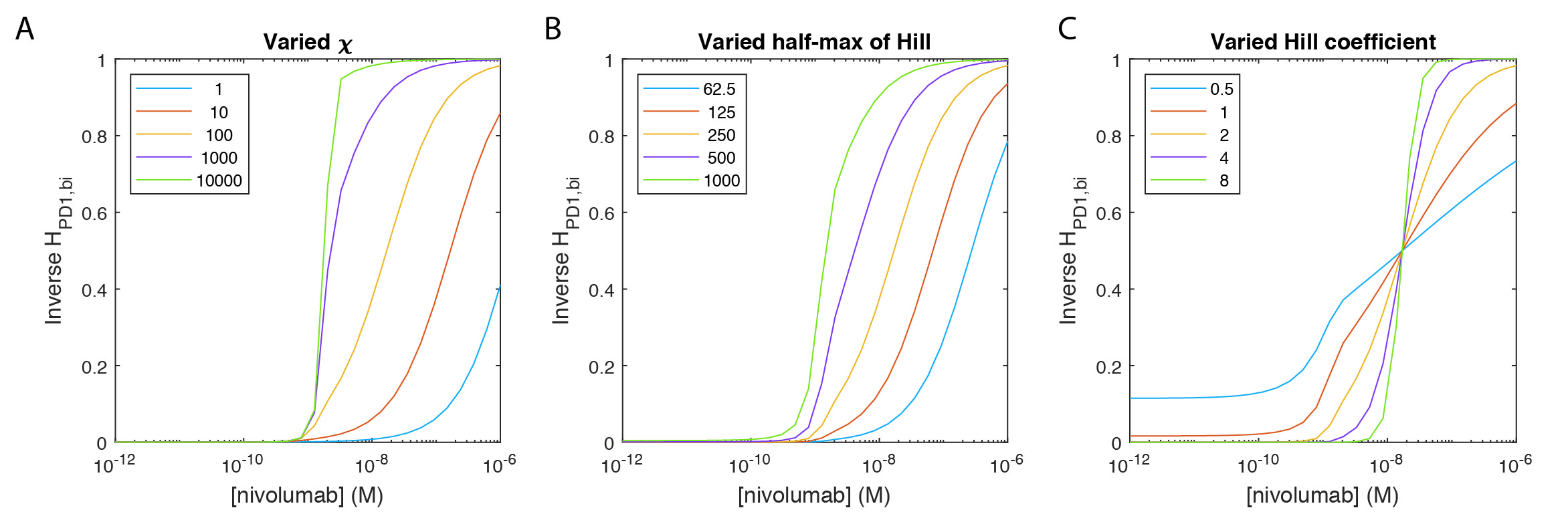


Figure S7. Comparison of effect of bivalent antibody model parameters in nivolumab dose response.

Unknown parameters affecting the antibody-mediated inhibition of the PD-1 interactions are varied to identify a reasonable assumption supported by the experimental data from Wang et al ([27](#_ENREF_27)). Three parameters of intrinsic antibody cross-arm binding efficiency ($X$), half-maximal activation and Hill coefficient of the Hill equation are varied. The yellow traces show the baseline case used in the model.
